# Engineered nanomedicine remodels the postoperative cavity microenvironment to suppress glioblastoma recurrence

**DOI:** 10.7150/thno.141774

**Published:** 2026-08-24

**Authors:** Zhiqun Bai, Zhiguang Chen

**Affiliations:** Department of Ultrasound, The First Hospital of China Medical University.

**Keywords:** glioblastoma, postoperative cavity microenvironment, smart responsive hydrogel, immunometabolic remodeling, ferroptosis

## Abstract

Glioblastoma (GBM) has a high rate of post-surgical recurrence, which can be attributed to residual tumor cells as well as to the disruption of the tumor microenvironment (TME) caused by surgery. Here, we define the postoperative cavity microenvironment (POCM) as a pathological niche with distinct spatial and temporal boundaries, and present a list of markers that outline this environment, including low pH, high levels of reactive oxygen species (ROS), high levels of glutathione (GSH), overexpression of matrix metalloproteinases, and tumor tissue mechanical instability. Engineered nanomedicine is evolving from simple drug-carrying entities to the design of smart responsive materials that can be actively tuned to modulate the POCM. In this review, we outline the working mechanisms and main features of different classes of smart responsive materials, which not only release therapeutic agents but also provide spatiotemporal control over drug delivery, enhance penetration into residual tumor tissue, and trigger immune activation at the right time and place. We discuss how local delivery of nanogel-based platforms in combination with systemic immunotherapy and fine-tuning by means of 3D bioprinting and/or microfluidic chip-based platforms can provide effective tools for reducing post-operative GBM recurrence rates.

## 1. Introduction

Glioblastoma (GBM) is the most common and aggressive primary malignant brain tumor in adults 1, 2. Despite the best available therapy including maximal safe resection followed by chemoradiation, the 5-year survival rate for GBM patients is less than 6% and the median overall survival is approximately 15 months 3. Residual tumor cells in the tumor resection bed are considered to be the sole cause of post-surgical relapse of gliomas, including GBM 4-6. However, in addition to residual tumor cells, local microenvironmental changes caused by the surgery itself can also trigger tumor relapse 7. The injury to the brain tissue caused by tumor resection leads to pathological responses that create a pro-recurrence niche in the surrounding tissue 8, 9. The time frame during which the pathological remodeling of the tumor resection cavity takes place is approximately 4 to 6 weeks after surgery during which time the cavity microenvironment is highly dynamic 9. The treatment gap for GBM patients between tumor resection and the start of standard chemoradiation therapy is a window of opportunity during which POCM-directed therapy can be offered to these patients. Thus, the concept of the postoperative cavity microenvironment (POCM) was developed. The POCM is a dynamic pathological niche shaped by surgical injury, BBB disruption, cellular debris, and residual tumor cells. For a period of time of approximately 4 to 6 weeks after surgery, the POCM can be delimitated spatially as the area of the resection cavity and the surrounding 2–5 mm tissue. It is therefore to be strictly differentiated from the post-operative microenvironment or “surgical microenvironment (SMe)” as described previously in the literature 10.

Nanomedicine for the treatment of the POCM represents a highly promising multidimensional approach to affect the tumor resection bed in glioma patients. POCM-directed nanomedicine can provide not only therapeutic benefit for glioma patients but also modulate the tumor microenvironment (TME) of GBM. Engineered hydrogels can be delivered to the resection cavity post-surgical tumor resection of the GBM patients as an injectable, sprayable, etc. formulation that fills the resection cavity with a nanoformulated hydrogel 12-15. Importantly, by filling the entire resection cavity with hydrogel that can conform to the surface of the resection cavity, a “smart” hydrogel nanomedicine formulation can deliver high concentration of chemotherapeutic agents to remaining glioma cells at the margin of the resection cavity POCM-directed hydrogel-based nano-formulations are being developed for delivery of a combination of chemotherapeutic drugs to eradicate remaining GBM cells after neurosurgical resection 16-19. Importantly, by co-delivering of combination of therapeutic agents that in conjunction with chemotherapeutic agent(s) (e.g., TMZ) deliver synergistic killing of stem cells that are considered to be responsible for GBM recurrence after brain tumor resection, including, for example, immune adjuvants, ferroptosis inducing drugs, etc., nanomedicine hydrogels can induce ferroptosis and/or autophagy-related death of GBM stem cells 20-22. Furthermore, by modulating POCM, nanogel deliver controlled drug release that can be modulated by changes in local tumor immune microenvironment over time, therefore, nanoformulations can be designed as “Stimuli-responsive nanogel composite system” for smart treatment with the possibility of on-demand drug release with temporal and/or spatial control 23-25. Additionally, a variety of new nano-immunomodulators for POCM-directed immuno-modulation therapy are under development that reprogram tumor associated macrophages (TAMs) in the TME of glioblastoma to display a pro-tumor immune reactive M1-like phenotype of TAMs instead of typical for GBM, immunosuppressive M2 TAMs in the tumor of GBM patients. Recently, a variety of Nano-Immunomodulators, including hydrogels, have been tested for induction of long-lasting antitumor-specific immune memory that is highly desirable in the treatment of tumors in general, including brain tumors, including the most aggressive and difficult-to-treat primary brain tumor, although the vast majority of brain tumors are gliomas 26,27. Thus, in this review, on the basis of PubMed/MEDLINE searches, Web of Science searches, Scopus searches, as well as based on evidence-based review of our previous work, we review current understanding of how multidimensional POCM nanoformulations can be successfully used to prevent recurrence of GBM in adult patients following brain tumor resection.

## 2. Key Pathological Features and Remodeling Targets of the POCM

Surgery removes the main tumor mass but also disrupts tissue homeostasis. The resulting wound-healing response can support repair of normal brain tissue and, at the same time, provide growth signals to residual GBM cells. The POCM should therefore be viewed as a changing niche rather than a static cavity. Its biology links immune suppression, metabolic adaptation, oxidative stress, and altered physical conditions.

### 2.1 Spatiotemporal dynamics and regional heterogeneity of the POCM

The rapid remodeling of the POCM to an immune suppressed tumor promoting environment is in direct conflict with the delayed start of post-surgical chemoradiation. The time it takes for patients to receive post-surgical chemoradiation is in itself sufficient to allow for the completion of immune reprogramming in the resection cavity prior to the initiation of adjuvant therapy. Indeed, Fernandes *et al*. 28 observed that fluorescently labeled liposomal nanoparticles injected intravenously within 15 minutes of tumor resection selectively accumulated in the resection margin tissue, whereas they were nearly undetectable in brain regions distant from the resection site, including brain tissue that was in direct contact with the resection cavity but was outside of the POCM. Furthermore, in a mouse model of post-operative GBM, Liu *et al*. 29 observed that macroscopic tumor recurrence occurred as early as 5 days post gross total resection (GTR), and was associated with early postoperative activation of the Akt pathway, and subsequent upregulation of tumor suppressor genes such as PD-L1, and the stem cell marker vimentin. The early POCM promotes a tumor promoting environment and represents a critical therapeutic window of opportunity.

**Figure 1:**
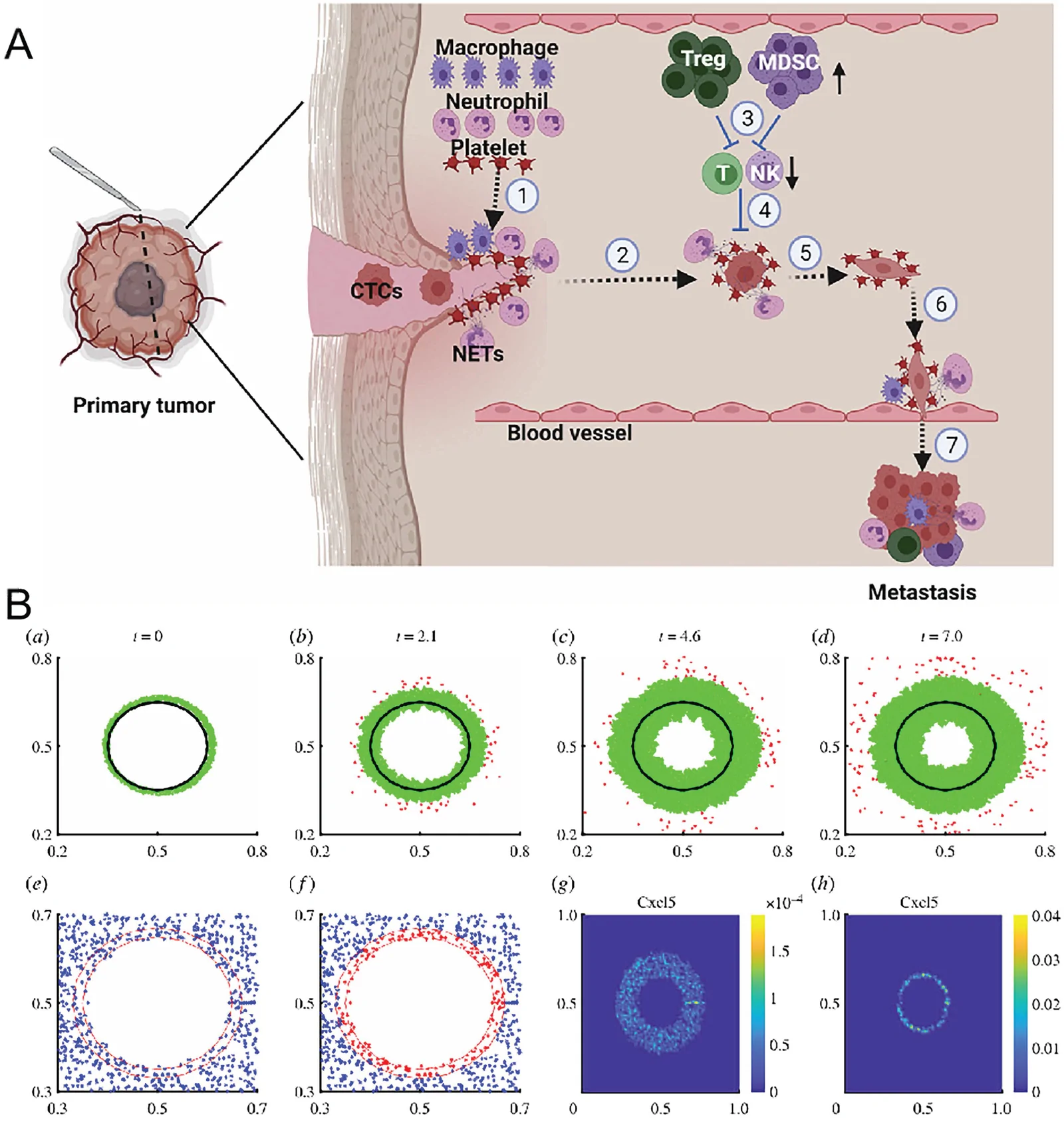
** Spatiotemporal dynamics and regional heterogeneity of the POCM. (A)** Surgery promotes formation of an immune microenvironment that supports tumor growth. Surgical injury recruits platelets, neutrophils, and alternatively activated M2 macrophages to the injury site for wound repair. Platelets and neutrophils protect circulating tumor cells (CTCs). Surgery increases immunosuppressive myeloid-derived suppressor cells (MDSCs) and regulatory T cells (Tregs), while suppressing natural killer (NK) cells and effector T cells capable of killing CTCs. Platelets promote epithelial-mesenchymal transition of CTCs. Together with M2 macrophages and neutrophils, platelets facilitate tumor-cell extravasation, after which CTCs colonize and expand at secondary sites. NETs denote neutrophil extracellular traps. Adapted with permission from Ref. 30. Copyright 2022 Elsevier Ltd. **(B)** Growth and invasion patterns of residual glioma cells after removal of the primary tumor center (a-d). Spatial distributions of reactive astrocytes and stem-like astrocytes at 1 h after surgery and at the terminal time point (e, f). Spatial distributions of Cxcl5 concentration at 0 h and 7 d after surgery (g, h). Adapted with permission from Ref. 32. Copyright 2020 the authors.

Postoperative resection margins represent heterogeneous areas with distinct molecular signatures. Cells within these margins behave differently from cells found in the primary tumor 9,30 (Figure 1A). Residual GBM cells located within resection margins are particularly hardy, resistant to cancer therapy, and exhibit an invasive phenotype 31,32 (Figure 1B). A considerable body of evidence demonstrated that, regardless of cancer treatment, local recurrence of GBM would arise from resection margins of tumors that were grossly resected 2, 26, 33. Cells located in resection margins, including GSCs, form stem-like, cancer stem cell- like populations by upregulating cancer stem cell- maintaining Notch1 signaling pathways 2. Surviving GBM cells may also utilize other signaling pathways to promote proliferation and migration by upregulating the Akt/PD-L1/vimentin pathway 28, 29. Cells located within resection margins are under the control of an immunosuppressive environment comprised of heterogeneous macrophages and polarized microglia that belong to the SMe and prevent destruction of residual tumor cells within the cavity 9, 34. The SMe can thus be viewed as having two major features: (1) a highly invasive subset of residual GBM cells within the SMe and (2) an immunosuppressive SMe that preserves residual tumor cells to allow their growth. It follows that the SMe is the true site of recurrence of GBM after treatment.

Key differences also exist with the post-operative microenvironment or SMe as typically defined and studied. Most notably, while studies of the post-operative microenvironment or SMe refer to any time point post-surgery without defining a temporal window 10, the POCM framework specifies a precise 0–6 weeks pre-adjuvant therapy time frame post-surgery. Secondly, the term ‘surgery-induced pro-tumorigenic microenvironment’ generally refers to changes in the microenvironment that are a consequence of surgery, but do not necessarily confine to the resection cavity and surrounding tissue 32. Importantly, the physical resection cavity plus surrounding 2–5 mm margin of tissue is the very same spatial distribution that harbors the bulk of residual tumor cells post-surgery, thereby being by far the most critical section of tissue post-surgery in terms of tumor relapse 2, 26, 33. Importantly, previous frameworks have largely considered the surgery-induced protumor microenvironment to be a ‘static’ construct that does not change with time 35, whereas POCM refers to a dynamic process (Table 1).

### 2.2 Immunosuppression and functional imbalance of the cellular landscape

#### 2.2.1 Polarization and Functional Reprogramming of Glioma-Associated Microglia and Macrophages

**Figure 2:**
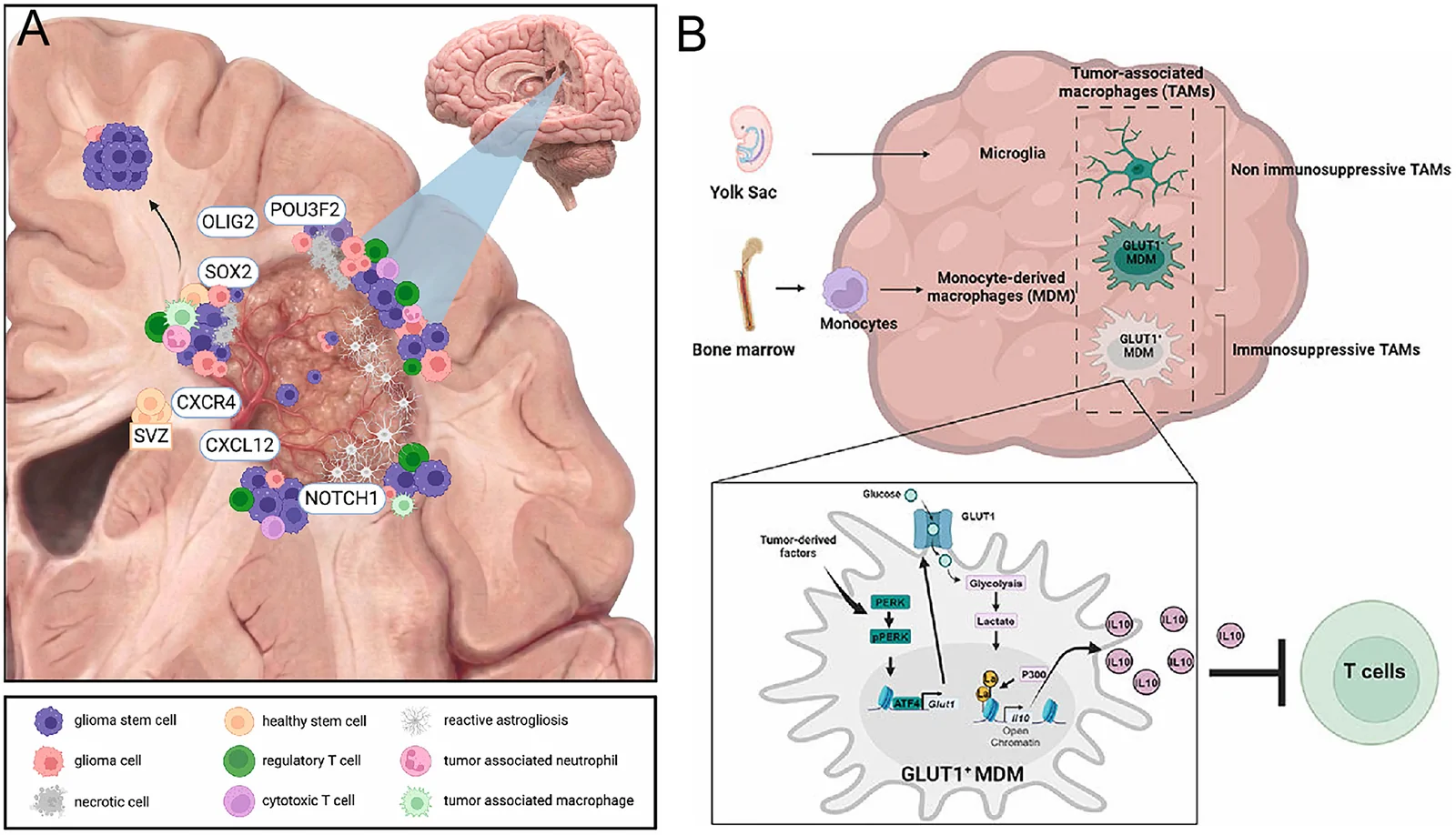
** Immunosuppression in the POCM. (A)** Cancer stem cell-driven local and distant recurrence in glioblastoma. The postoperative resection cavity illustrates that residual stem-like cell populations can persist after maximal resection combined with adjuvant therapy. These cells occupy multiple spatial compartments, including the infiltrative tumor margin, perivascular niches, hypoxic regions, and white matter tracts. The subventricular zone is considered a potential reservoir and site of dissemination initiation for tumor cells. Postoperative wound-healing signals may promote local tumor regrowth around the resection cavity and may also contribute to distant recurrence through remote dissemination. Adapted with permission from Ref. 9. Copyright 2026 the authors. **(B)** Glucose-driven histone lactylation promotes the immunosuppressive activity of monocyte-derived macrophages in glioblastoma. Adapted with permission from Ref. 40. Copyright 2024 Elsevier Inc.

Glioma-associated microglia and macrophages (GAMs) are the most abundant immune-cell population in the GBM microenvironment and are particularly enriched in the POCM. Studies have shown that reactive microglia and anti-inflammatory macrophages accumulate at the cavity margin early after surgery and become the dominant cellular populations at the onset of early recurrence 9,33 (Figure 2A). Driven by tumor-intrinsic signals and environmental cues, these cells undergo recruitment, polarization, and reprogramming to acquire tumor-supportive phenotypes.

Single-cell and spatial multi-omics studies have revealed the functional heterogeneity of GAMs and identified their reprogramming as a potential direction for POCM immunotherapy. Multiple studies indicate that local delivery of immune activators or gene-editing tools can reprogram GAMs from protumor to antitumor phenotypes. Shang *et al*. 20 devised a stimulator of interferon genes (STING) agonist prodrug hydrogel that promoted M1 polarization of GAMs, suppressed Treg activity, and increased cytotoxic T lymphocyte (CTL) infiltration. Wang *et al*. 36 created a lymph-node-inspired, acid-responsive hydrogel for siRNA delivery that targeted CD73 to reverse adenosine-mediated immuno-suppression. When combined with doxorubicin (DOX) and imiquimod, the hydrogel established a local inflammatory microenvironment, enhanced antigen presentation by GAMs, and promoted lymphocyte expansion. Chen *et al*. 37 and Fu *et al*. 38 induced the *in situ* generation of chimeric antigen receptor macrophages (CAR-Ms) within the resection cavity by delivering CAR genes and circular RNA, respectively. Engineered macrophages maintain proinflammatory polarization while being able to specifically recognize and phagocytose GSCs, thereby clearing postoperative residual tumors.

### 2.2.2 Treg accumulation and effector T-Cell exhaustion

There are extensive Treg infiltration but CD8+ T cells are in exhausted state in POCM. The post-radiation exosomes drive Treg expansion. The study by Tian *et al*. 39 demonstrated a novel mechanism of how irradiated GBM cells promote Treg expansion. Irradiated GBM cells release exosomes that are loaded with elevated levels of B7-H4 and deliver the molecule to naive Th cells to force the expression of FoxP3 during Th1 cell differentiation. Consequently, the normally tumor-aggressive Th1 cells are converted into suppressive Tregs that form immunosuppressive niches. Thus, in addition to promoting tumor growth, adjuvant radiation can also reinforce the immunosuppressive network and exacerbate the existing immunosuppression.

The surgical trauma alone is able to attract Tregs into the POCM, even in the absence of radiation, by inducing early Akt signaling following surgery to upregulate PD-L1 and vimentin in order to create a tumor promoting microenvironment that favors Treg persistence 29. This can be counteracted by the use of a self-assembling paclitaxel filamentous hydrogel in conjunction with an anti-CD47 antibody, which increases the phagocytosis by TAMs of the T cells by the tumor, resulting in enhanced activation of T cells. This formulation decreases the ratio of Tregs to CTLs, and provides a prolonged survival of mice bearing tumors following surgery 27. Another approach to counteracting the increase in Tregs in the POCM following surgery is to engineer a bacterial hydrogel superstructure that triggers pyroptosis in tumor cells. The bacterial components released from the hydrogel are able to recruit phagocytes and APCs, initiating adaptive immunity and causing a profound decrease in Treg suppressive function 33.

### 2.3 Lactate Metabolic Reprogramming and Histone Lactylation

Beyond acidifying the TME, lactate drives epigenetic reprogramming via histone lactylation, which alters immune cell signaling 41,42. This was also observed by De Leo *et al*. 40, who note that in large tumors there are many more infiltrating monocyte-derived macrophages (MDMs) than microglia. They also state that GBM-derived factors lead these MDMs to have very high rates of glycolysis, produce large amounts of lactate, and make IL-10. In such cases, intracellular lactate itself causes the lactylation of histones, increasing their tendency to express IL-10; hence, if MDMs inhibit T-cell activity, they must do so through IL-10 (Figure 2B). Then there is the PERK-ATF4 pathway. Tumor-derived factors activate it and thereby cause increased GLUT1 production in macrophages. Without PERK, no histone lactylation occurs in MDMs; thus, there will also be more T cells inside a tumor, less rapid tumor growth, and, finally, effective blockade of GBM progression if immunotherapy is administered on top. Hence, one can conclude from this study that it provides the first piece of evidence demonstrating that lactate-dependent epigenetic changes lead to immune inhibition by TAMs. Then there is another branch found by Wang *et al*. 43: lactate derived from GSCs taken directly from patients, along with some microglia and macrophages, induces epigenetic changes in cancer cells via histone lactylation. This turns on an immunosuppressive transcription program and increases levels of CD47, thereby stopping phagocytosis. Histones modified by lactate can bind to CBX3, one component of heterochromatin. CBX3 brings in EP300, an enzyme that adds acetyl groups to histone proteins. But CBX3 changes what EP300 prefers to work with: instead of acetyl-CoA, EP300 now uses lactyl-CoA. In such circumstances, genes encoding certain kinds of cytokines become more likely than others to be turned on. Then there is what happens inside the GBM microenvironment itself: TAMs release lactate outside their cells through an exporter called MCT4, and GSCs import this lactate via an importer known as MCT1. In fact, TAM-derived lactate serves two purposes here. First, it spurs GSC division, and second, it causes lysine residue 317 of KU70 in these cells to undergo lactylation. Hence, blockade of lactate transport or inhibition of KU70 lactylation along with ICIs may hold therapeutic promise 44.

### 2.4 Dysregulated cholesterol metabolism and lipid-laden macrophages

Cholesterol metabolism is another component of the immunosuppressive glioma niche. GBM cells rely heavily on extracellular cholesterol, whereas GAMs alter cholesterol synthesis, storage, and efflux. This interaction can generate lipid-laden macrophage states with functions that differ from those of conventional inflammatory or anti-inflammatory subsets.

**Figure 3:**
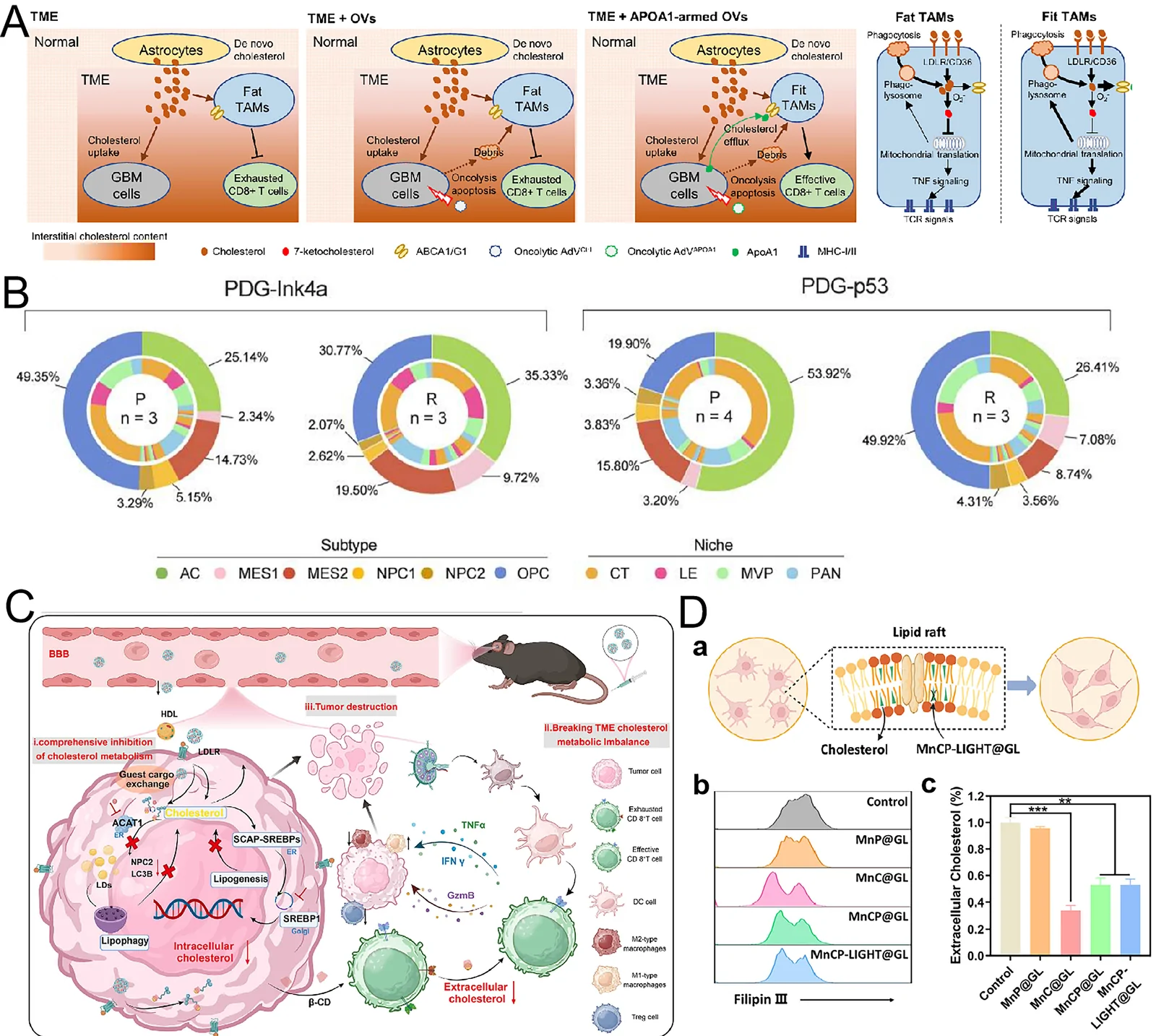
** Dysregulated cholesterol metabolism and lipid-laden macrophages. (A)** Macrophage plasticity in a cholesterol-rich microenvironment. Cholesterol accumulation impairs TAM phagocytosis by suppressing mitochondrial translation through 7-ketocholesterol. ApoA1-mediated cholesterol efflux restores TAM function and enhances TAM- and T-cell-mediated antitumor responses. Intratumoral delivery of an ApoA1-loaded oncolytic adenovirus remodels the immunometabolic microenvironment and promotes antitumor immunity. Adapted with permission from Ref. 45. Copyright 2023 the authors. **(B)** Mean proportions of glioblastoma cell subtypes in each model, shown in the outer rings. Pseudospatial allocation of glioblastoma cells, shown as subtype percentages in the inner rings. Adapted with permission from Ref. 46. Copyright 2024 Elsevier Inc. **(C)** A supramolecular nanoscavenger broadly corrects cholesterol imbalance in glioblastoma through a host-guest exchange mechanism while simultaneously remodeling the immunosuppressive TME. Adapted with permission from Ref. 47. Copyright 2025 Wiley-VCH GmbH. **(D)** Inhibition of GL-261 cell invasion by disrupting lipid rafts and suppressing lamellipodia formation (a). Intracellular cholesterol assay (b) and extracellular cholesterol assay (c). Adapted with permission from Ref. 48. Copyright 2025 Wiley-VCH GmbH.

Dong *et al*. 17 reported a distinct macrophage subset in GBM tissue and termed these cells glioma-supportive macrophages (GSMs). They found abnormally elevated cholesterol levels in GBM tissue, with GSMs functioning as major units of cholesterol synthesis and metabolism and exhibiting increased cholesterol metabolic and efflux activity. Wang *et al*. 45 further revealed a cholesterol metabolic codependence between GBM tumor cells and monocyte-derived TAMs. Tumor-cell demand for exogenous cholesterol was functionally coupled to impaired phagocytosis in TAMs caused by disordered cholesterol metabolism (Figure 3A). Promoting cholesterol efflux with apolipoprotein A1 (ApoA1) restored TAM phagocytic activity and reactivated antitumor immune responses mediated by the TAM/T-cell axis. Cholesterol metabolomics further showed that ApoA1-mediated lipid metabolic remodeling reduced 7-ketocholesterol levels. This metabolite directly suppresses tumor necrosis factor signaling in TAMs by blocking mitochondrial translation.

Cholesterol dysregulation may be further aggravated in the POCM. Surgical injury releases large amounts of cellular debris into the local microenvironment, and cholesterol-rich myelin debris provides an abundant lipid substrate for GSMs and other macrophages. Some TAMs acquire a lipid-laden phenotype by engulfing this myelin debris and then transfer myelin-derived lipids directly to cancer cells through an LXR/ABCA1-dependent pathway, thereby meeting the increased metabolic demands of mesenchymal GBM 46 (Figure 3B). Although postoperative models have not yet directly verified the formation of lipid-laden macrophages (LLMs) driven by myelin debris, several local therapeutic strategies have demonstrated the potential to suppress postoperative recurrence by targeting cholesterol metabolism.

Based on host-guest interactions between β-cyclodextrin and cholesterol, Tian *et al*. 47 devised a supramolecular nanoscavenger. The nanoscavenger competitively displaced and removed free cholesterol from the cholesterol-rich GBM microenvironment while providing controlled release of targeted drugs. It thereby corrected tumor-associated cholesterol dysregulation, inhibited tumor-cell proliferation and invasion, and promoted remodeling of the immunosuppressive microenvironment (Figure 3C). Qu *et al*. 48 created a biodegradable nanoregulator, MnCP-LIGHT@GL, loaded with cholesterol oxidase. This system directly oxidized and depleted cholesterol in the TME while generating reactive oxygen species (ROS), inducing pyroptosis and immunogenic cell death (ICD), and initiating a metabolic and immune cascade (Figure 3D).

Taken as a whole, these studies support cholesterol metabolism as a postoperative target, although the desired degree of depletion remains uncertain. LXR agonists promote GBM-cell death by inducing IDOL-dependent degradation of LDLR and increasing ABCA1-mediated efflux. HDL nanodiscs carrying an LXR agonist also prolonged survival when combined with radiotherapy 49. The evidence supports local cholesterol regulation, but it does not imply that indiscriminate cholesterol removal will be beneficial to every immune-cell population.

### 2.5 Oxidative stress and ferroptosis: from dual effects to therapeutic breakthroughs

ROS are normally produced by cellular metabolism. Low levels act as signals involved in cell division. Oxidative stress occurs if more is generated than the antioxidant capability allows. There can be increased ROS production within the surgical wound owing to damaged tissues and remaining tumour masses. It remains to be seen what effect this might have, because whether oxidative damage occurs will depend on how much ROS develop, how long they last, and the antioxidative status of the responding cells.

There is much excitement over chemodynamic therapy (CDT) and photodynamic therapy (PDT), which rely on very high levels of ROS to kill leftover cells altogether. However, sublethal ROS levels may instead activate NF-κB- and HIF-1α-dependent survival pathways, induce an EMT, and cause drugs to fail. In fact, Kim *et al*. 50 recently found that cannabidiol brought about more ROS via ERK signaling and thus caused autophagy and subsequently ferroptosis in glioblastoma multiforme cells. Conversely, according to Su *et al*. 51, CYBB-supported ROS kept the mesenchymal program going and prevented TMZ from having any effect, whereas Nrf2/SOD2 signaling protected against ferroptosis. Therefore, one cannot conclude merely from the amount of ROS itself whether cells will die or survive; antioxidant adaptation also needs to be considered.

Ferroptosis is one kind of RCD. It relies on intracellular Fe²⁺ to produce large amounts of ROS via the Fenton reaction; ROS then oxidizes polyunsaturated fatty acids in membrane phospholipids, triggers a chain reaction of lipid peroxidation, eventually causes irreversible damage to the membrane, and thereby leads to cell death 52,53. Nowadays, many scholars believe that promoting ferroptosis may become another effective method to cure GBM. In fact, several reports have shown that some drugs can induce ferroptosis in GBM cells by raising levels of ROS and lipid peroxidation, causing lysosomal membrane rupture, increasing radiotherapy sensitivity, etc. Disulfiram (an alcoholism medication) is one typical representative among these medicines 54.

Sensitivity to ferroptosis varies among GBM cell states. Ferroptotic cells also release DAMPs and oxidized products that can alter surrounding immune cells. In the study by Yang *et al*. 55, copper-selenium naphthoquinone nanoparticles induced both ferroptosis and cuproptosis, increased CD8⁺ and CD4⁺ T-cell infiltration, and extended median survival by 1.9-fold. The immune effect is nevertheless dose dependent. Excessive ferroptosis may worsen hypoxia, nutrient stress, or inflammatory immune suppression. Clinical use will therefore require control over where, when, and to what extent lipid peroxidation occurs.

### 2.6 Surgery-specific remodeling of the microenvironment

There are some similarities here: the POCM shows many of the same phenotypes as those found in the primary TME itself; these include suppression of immunity, changes in metabolism, and the presence of oxidants. However, they arise differently. In an intact tumor, such traits occur mostly because they result from continued signaling by tumor cells and also derive partly from prolonged immune escape. When the mass is removed, there is also sudden damage, which brings about breakdown of the BBB, wounding responses, discharge of broken pieces, and other mechanical factors that were not previously applicable (Table 2).

Therefore, on one hand, surgery destroys the structure of tissues locally while changing their hydrodynamics and creating certain kinds of mechanical constraints thereupon. During the first week after operation, the BBB will open temporarily. There is evidence showing that one may estimate the degree of such opening quantitatively. Indeed, some scientists have confirmed this point in both mice and people with GBM. Hence, this gives rise to a special opportunity for bringing nano-drugs into play. At the same time, however, wounds inflicted by surgeons abruptly initiate an intense pro-repair signal transduction pathway. Numerous neutrophils rush towards the resection cavity shortly afterwards. Then they produce many NETs themselves and thereby help leftover cancer cells multiply quickly and move out easily. In addition, reactive astrocytes acquire a protumoral type and release substantial amounts of CXCL5. Thus, they make more room available for gliomas to invade. Moreover, DAMPs emerging from widespread cell death provoke even stronger inflammatory reactions, while large amounts of cellular debris derived from surgical removal provide rich nutrients for macrophages. Especially noteworthy here is myelin debris that contains plenty of cholesterol. Afterwards, LLMs consume such remains but pass part of what they obtain from digestion over to mesenchymal-like glioblastoma cells through LXR/ABCA1. In this manner, the latter gain extra sources of lipids needed for metabolism; therefore, tumors continue growing because surgery causes another kind of metabolic adjustment.

There is a physical cavity, transient BBB permeability, NET formation, reactive gliosis, and myelin-debris recycling. Taken together, these features make the postoperative compartment different from the primary TME. The POCM is generated by surgical injury but is defined by the residual components and the altered local environment. Thus, recurrence may now also be thought of as growth occurring inside this novel kind of lesion rather than merely more of the old tumor continuing on.

## 3. Engineered Nanomaterials for Remodeling the Immune Microenvironment

### 3.1 Local delivery of immunomodulators

Local delivery makes it possible to expose the cavity to immunomodulators while limiting systemic distribution. CpG oligonucleotides, which activate TLR9 in antigen-presenting cells, are a well-established example 57,58. Zhang *et al*. 59 incorporated CpG nanoparticles and the CXCR4 antagonist Zn(II)₂-AMD3100 into the injectable imGEL hydrogel. AMD3100 reduced recruitment of microglia and macrophages, whereas CpG promoted cytotoxic T-cell activation. In the postoperative model, these two effects altered the local immune composition and reduced recurrence.

**Figure 4:**
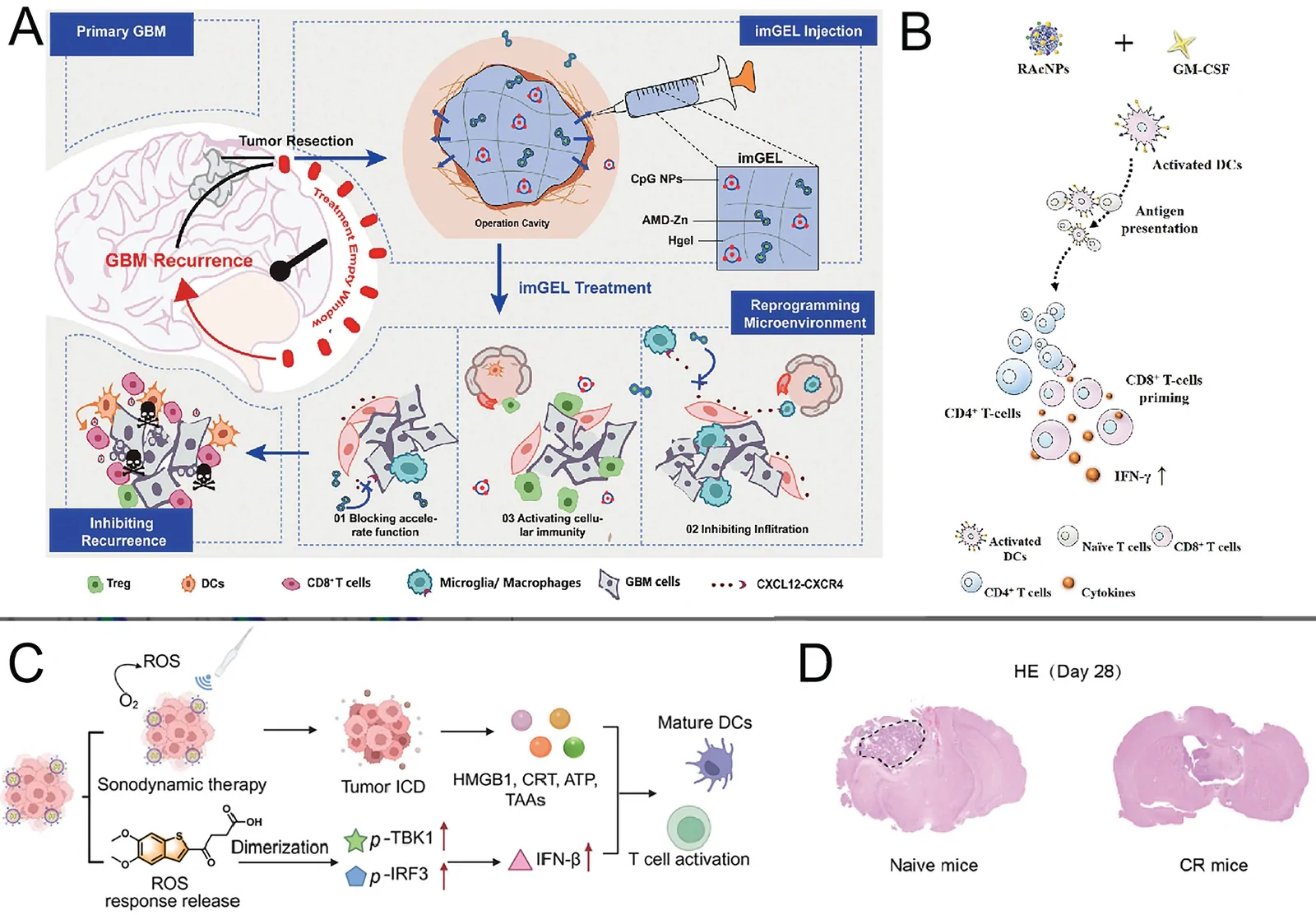
** Local delivery of immunomodulators. (A)** An immune-exosome platform that crosses the BBB for combined chemoimmunotherapy. Adapted with permission from Ref. 60. Copyright 2023 American Chemical Society. **(B)** A STING agonist prodrug hydrogel promotes M1 macrophage polarization, suppresses Treg activity, and enhances CTL infiltration. Adapted with permission from Ref. 20. Copyright 2026 the authors. **(C)** A sono-responsive nanoplatform integrating STING activation and CXCR4 blockade for synergistic immunotherapy. Adapted with permission from Ref. 61. Copyright 2025 Wiley-VCH GmbH. **(D)** The injectable ROS-degradable hydrogel ADU-AAV-PD1@Gel induces long-term immune memory through synergistic radioimmunotherapy. Adapted with permission from Ref. 63. Copyright 2022 the authors.

Cui *et al*. 60 developed the immune-exosome platform CpG-EXO/TGM. Self-assembled nano-micelles of tanshinone IIA and glycyrrhetinic acid were encapsulated in endogenous serum exosomes, and CpG was anchored to the exosomal membrane. After crossing the BBB, the platform was efficiently taken up by GBM cells and released its cargo to induce apoptosis. At the same time, CpG promoted dendritic-cell maturation and TAM polarization, generated an anti-GBM immune response, and prevented postoperative recurrence (Figure 4A).

Local activation of the STING pathway is now being studied more actively. Shang *et al*. 20 described a STING agonist prodrug hydrogel that formed an *in situ* drug depot within the resection cavity and continuously released therapeutic agents. The hydrogel promoted M1 macrophage polarization, suppressed Treg activity, and enhanced CTL infiltration, thereby reducing orthotopic GBM recurrence and establishing durable immune memory (Figure 4B). Kang *et al*. 61 developed a sono-responsive nanoplatform by co-assembling a high-performance sonosensitizer with a STING agonist prodrug and coating the assembly with glioma-cell membrane and a CXCR4-targeting peptide. Under ultrasound irradiation, the platform generated abundant ROS and synchronously released the STING agonist through a self-accelerating mechanism. The treatment activated innate and adaptive immunity while inhibiting the CXCL12/CXCR4 axis and reducing infiltration of immunosuppressive cells, thereby suppressing primary tumor growth and postoperative recurrence (Figure 4C).

Local delivery of immune checkpoint inhibitors is an important approach for reversing T-cell exhaustion in the POCM. Meng *et al*. 62 produced an *in situ* gelling fibrin drug-delivery system, PX-478 + αPD-1@Gel, co-loaded with the HIF-1α inhibitor PX-478 and an anti-PD-1 antibody. PX-478 alleviated the immunosuppressive microenvironment and enhanced T-cell infiltration by suppressing HIF-1α and PD-L1 expression, after which released αPD-1 further amplified the immune response. In an orthotopic postoperative glioma mouse model, the gel significantly reduced recurrence and prolonged median survival. Sun *et al*. 63 designed the injectable ROS-degradable hydrogel ADU-AAV-PD1@Gel, combining long-term AAV-mediated expression of soluble PD-1 with the STING agonist ADU-S100. Postoperative use in combination with radiotherapy induced long-lasting immune memory (Figure 4D). The studies above suggest that nanoplatforms capable of locally co-delivering multiple immunomodulators have become a general strategy for immunotherapy of the postoperative GBM resection cavity.

### 3.2 Regulation of macrophage polarization

**Figure 5:**
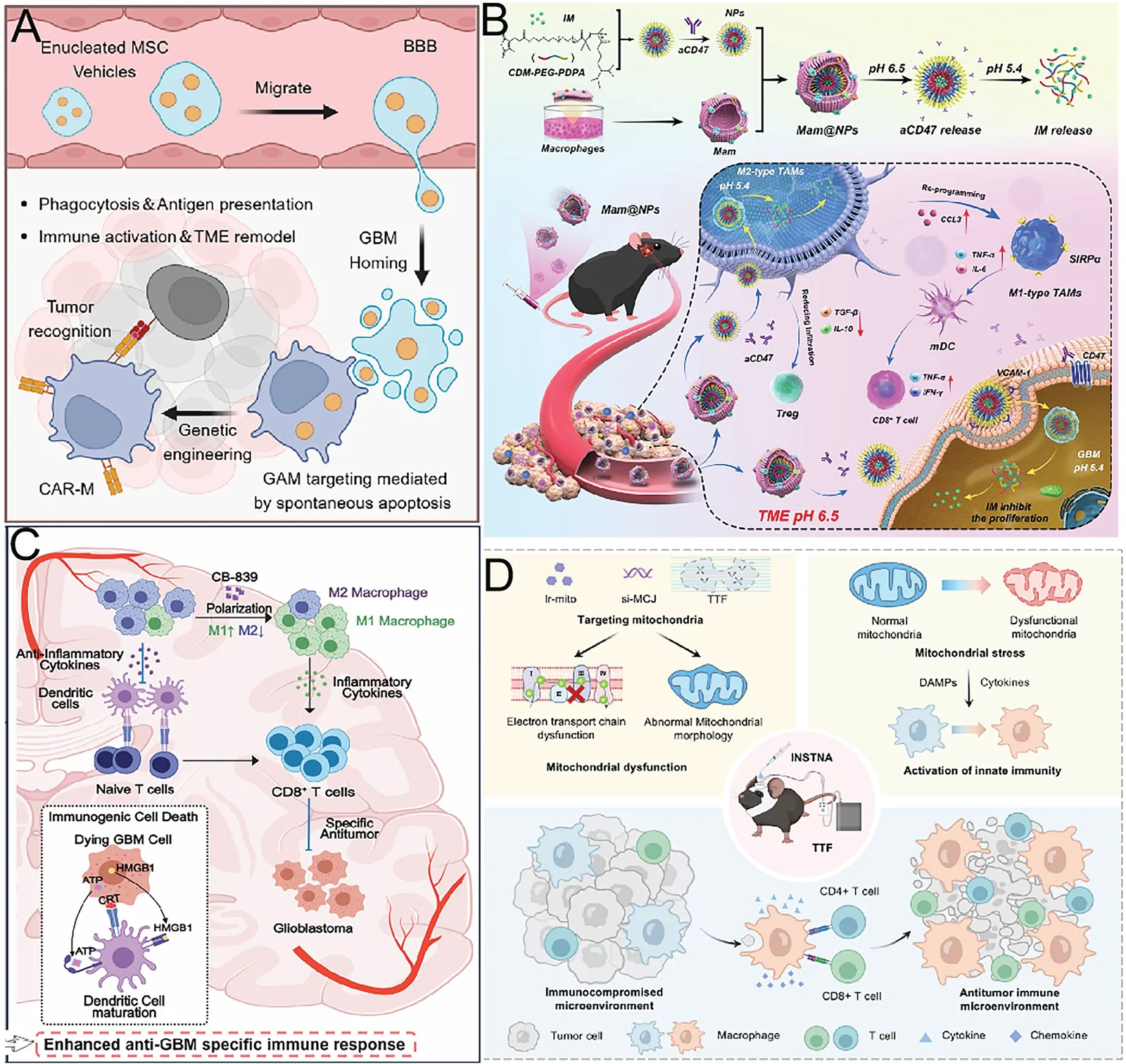
** Regulation of macrophage polarization. (A)** Enucleated mesenchymal stem cells serve as delivery vehicles for introducing CAR-encoding plasmids into GAMs, thereby generating CAR macrophages *in vivo*. Adapted with permission from Ref. 66. Copyright 2025 PNAS. **(B)** Released IM and aCD47 repolarize M2 TAMs toward the M1 phenotype and enhance the phagocytic activity of M1 TAMs. These cells subsequently secrete inflammatory and antitumor cytokines, promote DC maturation, stimulate CD8⁺ T-cell proliferation and activation, and reduce Treg infiltration. Adapted with permission from Ref. 27. Copyright 2023 PNAS. **(C)** Abundant ROS damage tumor cells and induce ICD. This process releases DAMPs, which trigger antitumor immune responses, promote DC maturation, and activate T cells. CB-839 simultaneously promotes polarization of M2 macrophages toward the M1 phenotype, thereby reprogramming the TME. Adapted with permission from Ref. 69. Copyright 2025 Wiley-VCH GmbH. **(D)** Immune-activated anti-GBM mechanism induced by innate immune-stimulating nanoparticles (INSTNAs) combined with tumor-treating fields. Adapted with permission from Ref. 71. Copyright 2025 Wiley-VCH GmbH.

GAMs are the dominant immune cell population inside the POCM, and their skew toward M2 polarization is what largely drives the immunosuppressive milieu. Nanomaterials can rewire GAMs from a pro-tumor phenotype to an anti-tumor one—through active targeting, cell-membrane mimicry, gene editing, metabolic modulation, or some combination of these. More recently, generating CAR-Ms directly *in situ* has emerged as a genuinely fresh approach 64,65. Delivering CAR genes to the nuclei of macrophages and microglia that reside in the resection cavity of brain tumors using an injectable nanoporous hydrogel superstructure enables the generation of GSC-specific CAR-Ms *in situ*. Chen and coworkers 37 in their innovative approach demonstrated that CAR-Ms not only were able to phagocytose GSCs, but also, upon GSC engulfment, could induce an adaptive antitumor immune response (Figure 5A). Subsequent studies that utilized other approaches (SIGLEC9-based chimeric switch receptor combined with a delivery system of ionizable lipid nanoparticles bearing bicistronic circular RNA that reprograms the signal from an inhibitory to an activating one for instance) demonstrated that even in a highly sialylated, and acidic TME, tumor-agnostic, pro-inflammatory, and phagocytic-reprogrammed macrophages can be successfully generated, thus enabling the establishment of an antitumor immune response, local as well as systemic 38. Importantly, long-term immune memory can be generated by repurposing, using an injectable nanogel system for instance, the generated macrophages around the resection cavity, thereby, enhancing tumor killing in subsequent challenges. Zhou and coworkers 66 in another innovative study built upon the previous work in the generation of CAR-Ms by employing enucleated mesenchymal stem cells (MSCs) for the *in situ* generation of CAR-Ms in the brain resection cavity using the MSCs as a delivery vehicle.

The use of macrophage membrane-biomimetic nanoparticles allows for homotypic targeting as well as signal regulation 67. Luo *et al*. 68 constructed the biomimetic delivery system SIRPα@BSA/PTX, which encapsulated albumin-bound paclitaxel and displayed a genetically engineered SIRPα variant on its surface. After crossing the BBB, the system synergistically promoted GAM repolarization and restoration of phagocytic function. When combined with an immune checkpoint inhibitor in a postoperative model, it produced 100% survival in mice. Wang *et al*. 27 formulated a self-assembling paclitaxel filamentous hydrogel and combined it with an anti-CD47 antibody to construct the aCD47/PF system. Paclitaxel fibers promoted formation of an immune-activated microenvironment and sensitized tumors to aCD47-mediated blockade of antiphagocytic signaling, thereby enhancing macrophage phagocytosis of tumor cells and activating T-cell responses (Figure 5B). Inspired by lymph-node function, Wang *et al*. 36 developed an acid-responsive hydrogel to deliver CD73-targeting siRNA and reverse adenosine-mediated immunosuppression. DOX simultaneously released tumor antigens, while imiquimod helped establish a local inflammatory microenvironment, substantially improving tumor clearance.

Regulation of macrophage polarization through metabolic reprogramming is increasingly being examined as a therapeutic strategy. Owing to high DHCR7 expression, GSMs synthesize and export large amounts of cholesterol, continuously supplying metabolic substrates for GBM growth while inducing CD8⁺ T-cell exhaustion 17. Guo *et al*. 69 devised an immunostimulatory hydrogel that reversed the immunosuppressive function of GAMs by blocking glutamine metabolism (Figure 5C). Guo *et al*. 70 created biomimetic membrane vesicles that selectively delivered iPERK to M2 microglia and reprogrammed them into M1 macrophages through the PERK/HIF-1α/glycolysis pathway, consistent with the PERK target described above in lactate metabolic regulation. Han *et al*. 71 prepared innate immune-stimulating nanoparticles loaded with an iridium complex and si-MCJ to induce mitochondrial stress. When combined with tumor-treating fields, these nanoparticles substantially remodeled the immunosuppressive microenvironment (Figure 5D).

Macrophage reprogramming cannot be considered in isolation from the other treatments placed in the cavity. Ferroptosis induction and cholesterol depletion may either reinforce or interfere with macrophage activation, depending on dose and sequence. Combination design therefore requires direct testing of these interactions rather than assuming that two individually active components will be additive.

### 3.3 Induction of ICD and *in situ* vaccination

ICD is a form of regulated cell death that spills damage-associated molecular patterns and tumor antigens, turning dying tumor cells into an *in situ* vaccine—they rouse dendritic cells and kick-start adaptive immunity 72. Inside the POCM, using nanomaterials to push residual tumor cells into ICD is a core strategy for converting an immune-cold tumor into a hot one.

**Figure 6:**
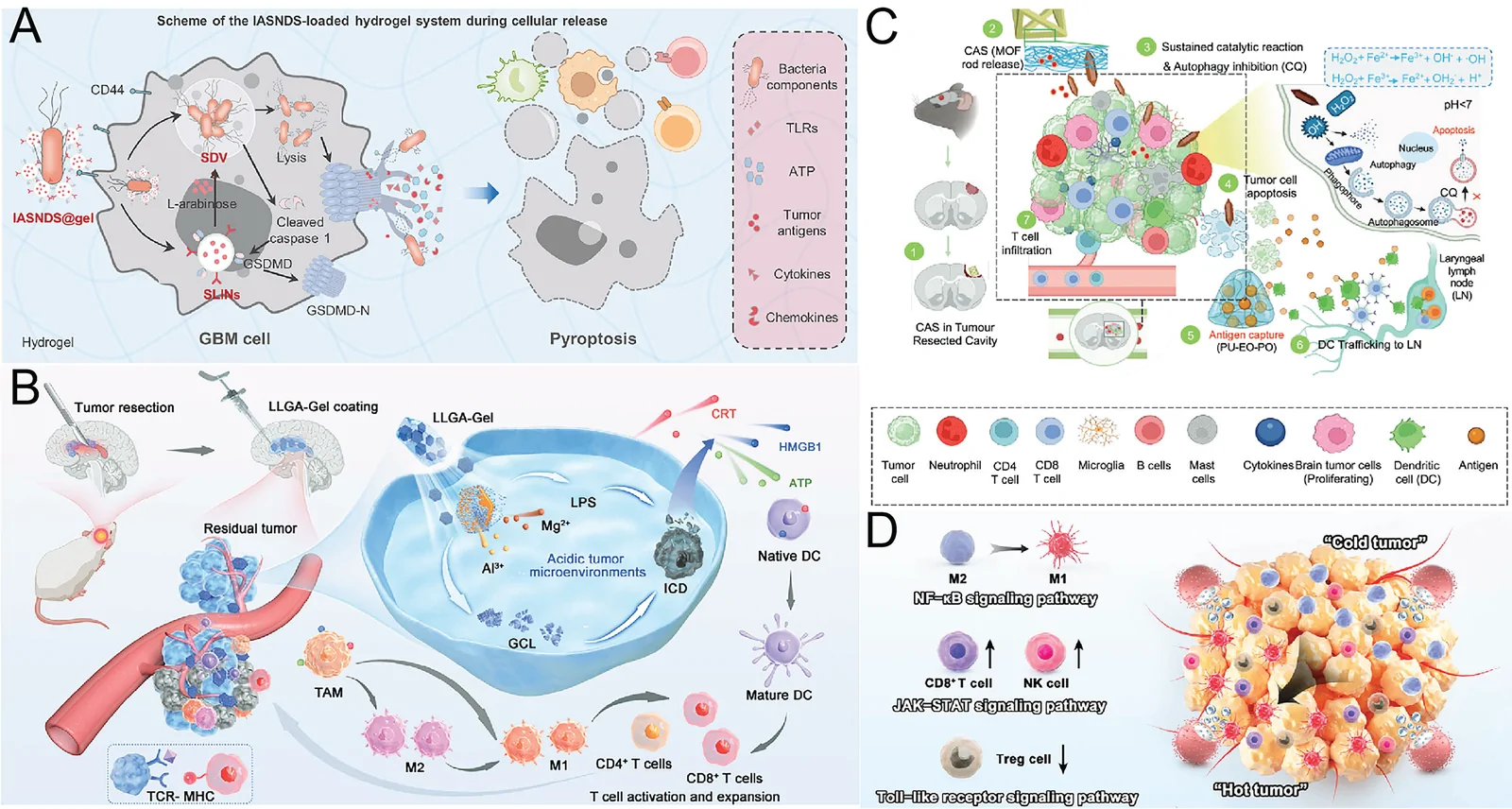
** ICD induction and *in situ* vaccination. (A)** A bacterial hydrogel superstructure enables bacterial entry into cells, initiates intracellular autolysis, and induces tumor-cell pyroptosis. Adapted with permission from Ref. 33. Copyright 2023 the authors. **(B)** An *in situ* sprayed exosome-crosslinked gel functions as an artificial lymph node for postoperative glioblastoma immunotherapy. Adapted with permission from Ref. 75. Copyright 2024 American Chemical Society. **(C)** A self-cascading catalytic therapy and antigen-capture scaffold releases catalytic therapeutic agents from MOF rods and serves as an antigen reservoir to promote postoperative brain-tumor immunotherapy. Adapted with permission from Ref. 76. Copyright 2024 Wiley-VCH GmbH. **(D)** Mg-Motor-DOX is uniformly dispersed in a thermosensitive hydrogel formed from F127 and F68. After partial GBM resection, Mg-Motor-DOX@Gel is injected into the resection cavity, crosslinks *in situ*, and covers the surgical region. Within the GBM microenvironment, MMP-2 overexpression promotes DOX release from Mg-Motor-DOX, while reaction of magnesium with water releases H₂. H₂ reduces inflammation in the surgical region and enhances active DOX delivery, thereby improving DOX efficacy through hydrogen-chemotherapy synergy. Adapted with permission from Ref. 77. Copyright 2025 Wiley-VCH GmbH.

Pyroptosis and bacterial therapy are two powerful ways to ignite robust ICD. Zhang *et al*. 33 used attenuated Salmonella as an immunostimulatory delivery chassis, coupled it with nanocapsules that trigger bacterial autolysis, and built an injectable bacterial hydrogel superstructure. This construct homes to residual GBM satellite foci inside the resection cavity, drives pyroptosis in the tumor cells, and releases bacterial components that call in phagocytes and APCs—activating both innate and adaptive immunity and effectively holding post-surgical recurrence in check (Figure 6A). Yang *et al*. 73 took a different route: they co-loaded lipopolysaccharide (LPS) and GBM cell lysate onto layered double hydroxide nanosheets and embedded them into an alginate hydrogel to create an autologous nanovaccine. The immunostimulatory punch of LPS works in lockstep with the immunogenicity of the lysate, prompting the vaccine to trigger pyroptosis, boost DC maturation, shove macrophages toward M1 polarization, ramp up CD8⁺ T-cell infiltration, and dial down Foxp3⁺ Tregs.

ICD driven by ferroptosis and cuproptosis has drawn sharp attention in recent years. Guo *et al*. 69 crafted an injectable peptide hydrogel that co-delivers the glutaminase inhibitor CB-839 and self-assembled copper-peptide particles. To drive GBM cells into ICD (and thereby change the tumor’s immune microenvironment to suppress recurrence and extend the median survival of the animals), by amplifying copper-peptide-mediated CDT through inhibiting glutamine metabolism for driving up intracellular oxidative stress, Zhang *et al*. 74 designed an albumin-based nanodelivery system, BSA@CTW-NPs, which contained wogonin and TMZ and introduced Cu²⁺ to generate oxidative stress via a Fenton-like reaction. BSA@CTW-NPs delivered their drugs not only systemically before surgery but also locally, at the resection cavity, *in situ*, immediately after surgery, as a hemostatic sponge. Treatments with BSA@CTW-NPs, *in situ*, triggered cuproptosis and ICD in tumor cells and evoked a powerful antitumor immune response to suppress post-surgical tumor recurrence.

*In situ* artificial lymph node strategies add an innovative platform for amplifying T-cell activation on top of ICD. Bao *et al*. 75 harnessed a bioorthogonal reaction between azide-decorated chimeric exosomes and alkyne-modified alginate to design an *in situ* sprayable exosome-crosslinked gel. The chimeric exosomes are derived from dendritic cell–tumor cell fusions. The gel acts as a surrogate lymph node, directly activating tumor-infiltrating T cells, sidestepping rapid immune clearance, and prolonging antitumor T-cell immunity. Pair it with a STING agonist and the T-cell response intensifies—residual post-surgical lesions are nearly wiped out (Figure 6B). A self-cascading catalytic therapy and antigen-capture scaffold, developed by Yalamandala *et al*. 76, comprises of an iron-based metal–organic framework (MOF) that is released and undergoes Fenton reaction after getting converted to Fe²⁺. Tumor antigens, which are released from the scaffold made of polyurethane segments, are captured and presented to the DCs by the scaffold to bring about ICD (Figure 6C). Zhang *et al*. 77 deployed magnesium micromotors (Mg-Motor-DOX) to generate hydrogen gas right inside the resection cavity. The hydrogen propels the micromotors, eases neuroinflammation through antioxidation, and generates vortex flow to improve DOX penetration and GBM cell sensitivity. RNA sequencing confirmed that the strategy remodels the immune microenvironment by turning an immunologically cold tumor hot (Figure 6D).

### 3.4 Synergy and antagonism among multidimensional remodeling strategies

The strategies discussed above are often presented as mutually reinforcing. That assumption is not always justified. Immunomodulators, macrophage-directed agents, metabolic interventions, and cell-death inducers can produce synergy, but they can also oppose one another or increase toxicity when released in the same cavity. Three examples of these conflicts are considered below.

#### 3.4.1 Dual effects of ferroptosis

Ferroptosis can activate DCs and initiate antitumor immunity through DAMP release and is considered an important mechanism of *in situ* vaccine formation in the POCM. Recent studies, however, have revealed contradictory effects of ferroptosis on tumor immunity. Excessive or poorly controlled ferroptosis may instead promote immunosuppression. In a systematic study across several syngeneic murine tumor models, Mbah *et al*. 78 observed that ferroptotic tumor cells released immunostimulatory DAMPs also secreted immunosuppressive metabolites and oxidized phospholipids. Treatment with conditioned medium from ferroptotic cells substantially inhibited CD8⁺ T-cell proliferation and cytotoxicity. *In vivo* experiments further confirmed that inoculation with ferroptotic cells caused substantial accumulation of immunosuppressive myeloid cells within tumors, accompanied by reduced CD8⁺ T-cell infiltration.

At the molecular level, ferroptotic cells can release GPX4, which acts as a specific immunosuppressive DAMP. Binding of GPX4 to the ZP3 receptor on DCs inhibits DC maturation and weakens antitumor immune responses 79. Among tumor-associated immune cells, regulatory T cells depend on FSP1 to resist ferroptosis and maintain their immunosuppressive function. Targeting FSP1 selectively disrupts lipid-peroxide homeostasis in intratumoral Tregs and enhances antitumor immunity 80. Moderate induction of ferroptosis may therefore selectively eliminate Tregs, whereas excessive ferroptosis may aggravate immunosuppression through abundant release of inhibitory DAMPs such as GPX4. Overall, the response follows a characteristic bell-shaped dose-response relationship.

#### 3.4.2 Dual effects of cholesterol depletion

GBM-derived GSCs show an increase in DHCR7 expression which enables excessive cholesterol production and efflux. In tumor cells this can serve as a metabolic substrate, in CD8+ T cells, however, it can lead to exhaustion 17. However, cholesterol also serves positive roles in T cells by supporting the function of T cells in several ways, including being part of the T-cell receptor (TCR) signaling complex (lipid rafts), enabling nanoscale clustering of the TCR, increasing the affinity of the TCR for antigen and, overall, increasing the sensitivity of T cells 81.

Note that while TCR signaling will drop to very low levels of antitumor activity when cholesterol is depleted to extremes, on the other hand, high amounts of cholesterol in the TME will allow for the upregulation of immune checkpoint molecules to promote the exhausted state of CD8+ T cells 82. Therefore, there is a ‘bell-shaped curve’ of how cholesterol affects the activity of antitumor CD8+ T cells, that will need to be very accurately managed in POCM strategies that target cholesterol metabolism in the tumor and GSCs to decrease their levels and increase the levels of available cholesterol for antitumor CD8+ T cells. Ideally, such a strategy would remove excess cholesterol from tumor cells and GSCs, while either leaving T-cell cholesterol unchanged or even increasing it.

#### 3.4.3 Long-term safety of hydrogel degradation products and nanomaterial retention

And then there is what happens beyond the breakdown products themselves. Here another concern arises about the long-term persistence of some forms of inorganic nanoparticles once they have served their purpose in causing cell death. Certain kinds of nanoparticles seem designed expressly for this very reason—they consist largely of iron compounds or copper salts. Normally, one expects microglia-derived extracellular vesicles to take care of removing both kinds of particles from the body, whether organic or not. But when the debris proving difficult to dispose of happens to contain pieces of an inorganic substance, those remnants interfere with ERK1/2 signaling. As a result, fewer vesicles are produced, leaving too few to carry away the remaining traces of nanoparticles stuck within microglial cells and preventing the rest from leaving via normal channels provided by perivascular glymphatics 85. Hence, here again, some types of inorganic nanoparticles could remain lodged indefinitely afterwards. Gold nanoparticles still linger; quantum dots refuse to budge; iron oxides stay put; others refuse to leave; and thus, they continue causing damage later on. To solve this problem, pharmacologically stimulating ERK1/2 activity induces microglia to secrete more vesicles and increases the chance that some will contain one kind of troublesome nanoparticle or another. When scientists tried this treatment on experimental animals, they observed that roughly 5.48-fold as many quantum dots came out afterwards and nearly 28.56-fold more gold nanoparticles escaped through the usual routes. Therefore, in theory at least, one may be able to find ways of helping such substances depart sooner if necessary.

And again, one finds the glial reaction to hydrogels in terms of their degradation kinetics. Bjugstad *et al*. 86 implanted poly(ethylene glycol) (PEG) hydrogels with various kinds of mass-loss curves and examined how many microglia and astrocytes appeared in their vicinity over time. In this case, however, the hydrogel helped suppress the initial burst of microglia seen within the first week; but if there was no dissolution (or very slow dissolution), then microglial counts remained high through week eight, whereas they returned to lower levels later in controls without implants. And although the ultimate degree of astrogliosis turned out to be less than that resulting from merely poking the brain with a needle, degradation rate once more proved important for controlling inflammation in the end.

## 4. Metabolic Remodeling: From Cholesterol Regulation to Ferroptosis Induction

Dysregulated cholesterol metabolism is an important component of POCM immunosuppression. Owing to high DHCR7 expression, GSMs in GBM synthesize and export large amounts of cholesterol, providing metabolic substrates for residual tumor cells and directly inducing CD8⁺ T-cell exhaustion 17. This finding suggests that cholesterol metabolism can be regarded as a metabolic immune checkpoint and supports subsequent ferroptosis-induction strategies.

Following this rationale, Tian *et al*. 47 described a tumor-in-situ-triggered, cargo-exchange supra-molecular nanoscavenger. By exploiting the natural affinity between β-cyclodextrin and cholesterol, the system removed excess cholesterol from the cholesterol-rich GBM microenvironment, suppressed CD8⁺ T-cell exhaustion, and improved local immunosuppression. Halseth *et al*. 49 developed synthetic high-density lipoprotein (sHDL) nanodiscs for co-delivery of the LXR agonist GW3965 and CpG oligonucleotides. The nanodiscs depleted intratumoral cholesterol stores by upregulating expression of the cholesterol efflux transporter ABCA1. After combination with radiotherapy, 66% of long-term surviving mice remained tumor-free after tumor rechallenge.

**Figure 7:**
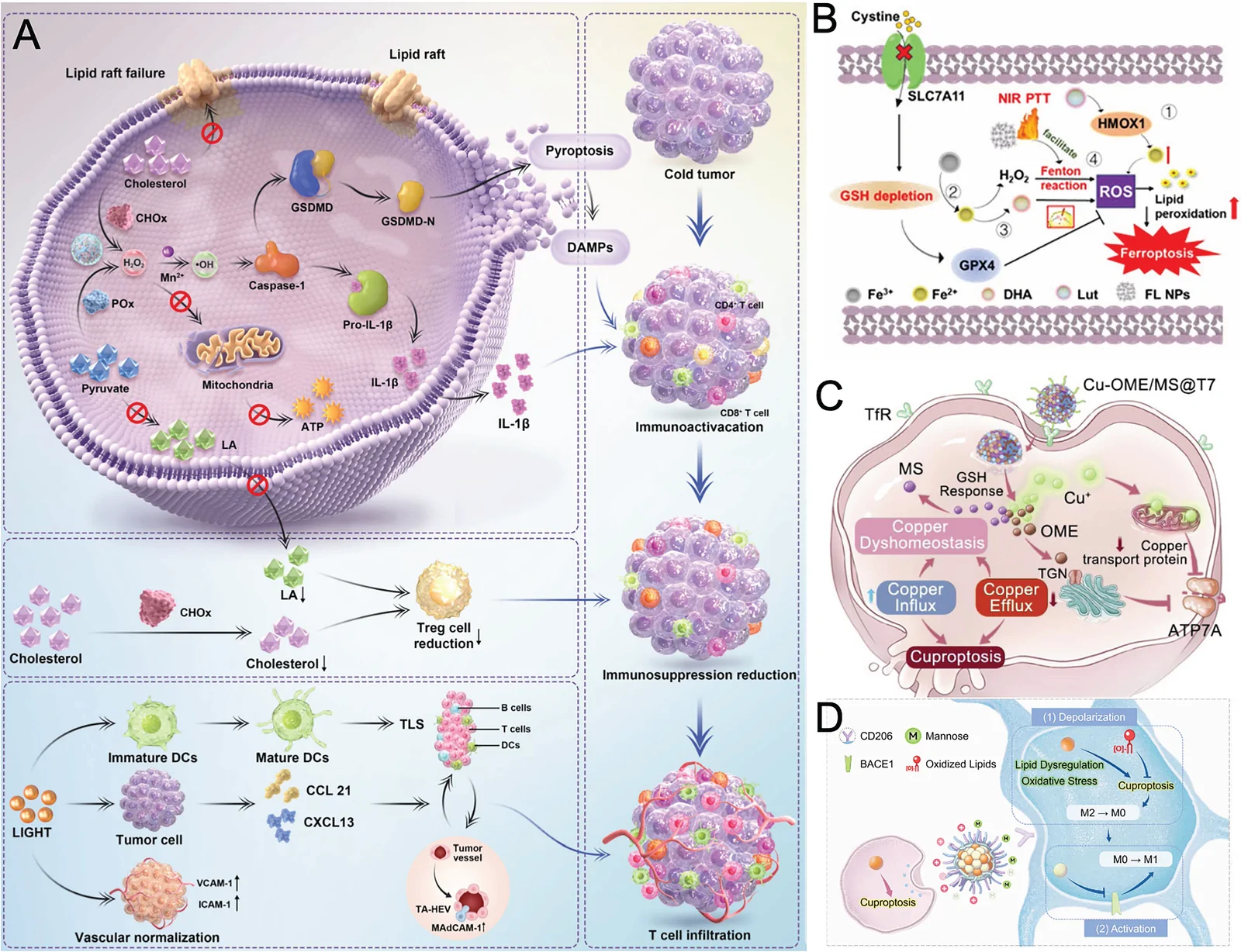
** From Cholesterol Regulation to Ferroptosis Induction: Metabolic Reprogramming. (A)** A biodegradable nanoregulator for glioblastoma immunotherapy. The nanoregulator was prepared through the biomineralization of Mn²⁺ with cholesterol oxidase and pyruvate oxidase and was loaded with the cytokine LIGHT. Its surface was coated with a glioma cell membrane. This design enables tumor targeting, induces pyroptosis, remodels immunosuppressive metabolism, and enhances antitumor immune responses. Adapted with permission from Ref. 48. Copyright 2025 Wiley-VCH GmbH.** (B)** FLD nanoparticles trigger ferroptosis. Adapted with permission from Ref. 89. Copyright 2025 the authors. **(C)** An omeprazole-mediated copper nanodelivery system simultaneously regulates copper influx and efflux for cuproptosis-based glioblastoma therapy. Adapted with permission from Ref. 94. Copyright 2025 Elsevier Inc. **(D)** EsCu/MK@M-P prepared using the FNP method specifically targets tumor lesions and microglia. Adapted with permission from Ref. 96. Copyright 2026 Wiley-VCH GmbH.

Qu *et al*. 48 produced the MnCP-LIGHT@GL nanoregulator through biomineralization of Mn²⁺, cholesterol oxidase, and pyruvate oxidase. This nanoregulator simultaneously targeted cholesterol and pyruvate metabolic pathways, reduced accumulation of immunosuppressive metabolites within the TME, and triggered pyroptosis through an Mn²⁺-mediated Fenton-like reaction (Figure 7A). The palladium-doped Cu₃N nanozyme platform Cu₃PdN@CR combined cholesterol depletion with ferroptosis and cuproptosis. Cholesterol oxidation induced metabolic stress and downregulated PD-L1 expression, while ultrasound stimulation amplified ROS generation to initiate ICD 87. Together, these strategies constitute an integrated regulatory cascade spanning from metabolic immune checkpoint blockade to the induction of cell death.

Important progress has also been made in therapies targeting ferroptosis. Zhang *et al*. 88 constructed iron oxide nanoparticles, FA/Pt-si-GPX4@IONPs, for co-delivery of cisplatin and GPX4 siRNA with folate receptor-specific targeting of GBM cells. Within this system, iron ions generated ROS through the Fenton reaction to induce ferroptosis, cisplatin promoted apoptosis, and si-GPX4 silenced GPX4, producing synergistic enhancement through three pathways. Wu *et al*. 23 described a postoperative injectable dual-network hydrogel preloaded with triptolide, TP@DNH. The hydrogel inactivated GPX4 by suppressing the NRF2/SLC7A11/GPX4 axis while generating ROS through a Fenton reaction catalyzed by an Fe³⁺/tannic acid metal-phenolic network, thereby promoting lipid-peroxide accumulation through two pathways. A luteolin-coordinated ferric-ion nanoplatform achieved quadruple synergistic amplification of ferroptosis by suppressing SLC7A11, reducing GSH synthesis, and decreasing GPX4 activity 89 (Figure 7B). Lactoferrin nanoparticle-vanadium complexes, LF-V4 NPs, induced ferroptosis through ROS accumulation, GSH depletion, and downregulation of GPX4 and SLC7A11 90, providing diverse nanodelivery options for targeting GPX4. Jia *et al*. 91 documented a lipoic acid-iron hydrogel, LFH, with mechanical strength compatible with brain tissue. After injection into the resection cavity, this hydrogel did not induce glial scar formation. Instead, cyclic redox reactions involving LA/DHLA and Fe³⁺/Fe²⁺ continuously generated ROS, promoted ferroptosis, and activated ICD. Additional loading with anti-PD-L1 enhanced the immunotherapeutic effect.

Cuproptosis is a recently identified form of copper-dependent cell death that can act synergistically with ferroptosis and enhance immune activation 92,93. Yang *et al*. 55 described copper-selenium naphthoquinone nanoparticles (CSN NPs) that were slowly released from a postoperative hydrogel. These nanoparticles triggered cuproptosis by aggregating mitochondrial lipoylated proteins and disrupting iron-sulfur cluster stability, while simultaneously inducing ferroptosis through a Fenton-like reaction and GSH depletion. The combined modes of death generated strong ICD and significantly increased median survival by 1.9-fold. Feng *et al*. 94 developed the copper-omeprazole supramolecular nanodrug Cu-OME SNDs, which disrupted copper homeostasis by synchronously regulating copper influx and efflux. Omeprazole (OME) coordinated with copper ions to form a self-delivering nanodrug and promote copper influx, while released OME inhibited the copper efflux transporter ATP7A and thereby remodeled copper export. After modification with a T7 peptide and minoxidil sulfate, the nanodrug crossed the BBB and established a positive-feedback loop (Figure 7C). RAP-anchored copper-escorting liposomes, RAP-LPs@ESCu, selectively targeted and eliminated metabolically active GBM cells through an FDX1-dependent cuproptosis pathway, providing direct evidence that FDX1 is a key cuproptosis target 95. Liu *et al*. 96 proposed a depolarization-activation strategy in which cuproptosis drove TAMs into a functional clearance phase and increased their sensitivity to immune activators, thereby establishing an innovative link between cuproptosis and immune resensitization (Figure 7D).

Cholesterol regulation, ferroptosis, and cuproptosis can be linked within a single metabolic treatment scheme, but each component has a different risk profile. Local placement gives hydrogels and nanofibers direct access to the cavity. GBM cells also have metabolic dependencies that may make them vulnerable to cholesterol withdrawal and lipid peroxidation. In addition, ferroptotic or cuproptotic death can provide antigens and danger signals that support checkpoint blockade.

## 5. Intracerebral Clearance and Metabolic Fate of Nanoparticles

Beyond therapeutic efficacy, the safety of nanoparticle-based POCM therapies depends critically on their clearance from the brain. This is particularly relevant to iron- and copper-based systems designed to induce ferroptosis or cuproptosis. Organic particles are often removed relatively quickly, whereas inorganic particles may persist and follow different clearance kinetics. Prolonged retention increases the possibility of chronic cellular stress or neurotoxicity 85.

The perivascular glymphatic pathway is the main route for nanoparticle clearance from the brain and accounts for approximately 80% of total clearance 97,98. Cerebrospinal fluid enters the brain parenchyma along periarterial spaces and exchanges with interstitial fluid. It is then drained through perivenous spaces 99. Organic nanoparticles, including reconstituted high-density lipoprotein and PEG-PLA nanoparticles, can be rapidly cleared through this pathway. Their half-lives are usually no longer than 5 h 97. However, the efficiency of this pathway is reduced under pathological conditions associated with glymphatic dysfunction, such as Alzheimer’s disease.

Microglia are resident immune cells of the central nervous system and participate in nanoparticle clearance through several mechanisms 100. Complement C3 opsonization activates a complement-dependent pathway that allows microglia to directly phagocytose nanoparticles. Microglia-derived extra-cellular vesicles can also promote the clearance of both organic and inorganic nanoparticles. However, inorganic nanoparticles can inhibit ERK1/2 signaling and reduce extracellular vesicle production. This increases intracellular nanoparticle accumulation and limits their drainage through perivascular pathways 85.

These differences prolong the retention of inorganic nanoparticles in the brain, including iron oxide nanoparticles, gold nanoparticles, and quantum dots. They may also increase the risk of neurotoxicity. After uptake by microglia, nanoparticles are first sorted in endosomes and then transported to lysosomes. Lysosomal hydrolases degrade them under acidic conditions at pH 4.5 to 5.0. The degradation rate depends on the chemical composition and surface properties of the nanomaterial.

**Figure 8:**
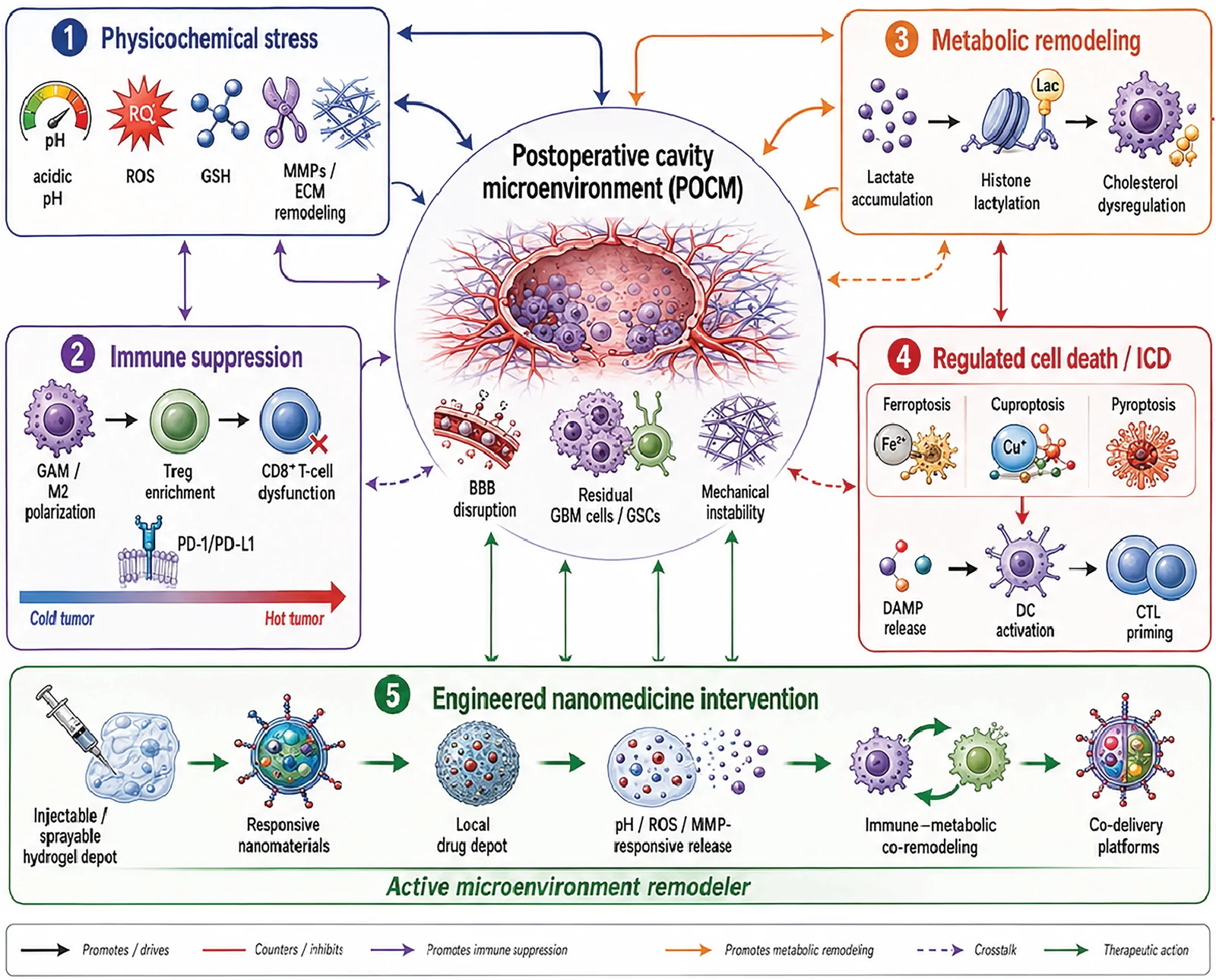
** Interaction networks in the POCM and engineered nanomedicine intervention strategies.** This schematic illustrates the interactions among physicochemical signals, immune remodeling, metabolic reprogramming, and regulated cell death in the POCM. M2 macrophage polarization, Treg enrichment, and metabolic stress form a positive feedback loop. Together, these processes support the survival of residual GBM cells and GSCs. They also promote local invasion, treatment resistance, and tumor recurrence. The lower part of the figure summarizes engineered nanomedicine intervention strategies. These include injectable or sprayable hydrogel drug reservoirs, pH-responsive, ROS-responsive, and MMP-responsive nanomaterials, ROS and GSH scavengers, nanozymes, and co-delivery systems. These platforms function not only as drug carriers but also as active regulators of the TME. By targeting multiple pathways simultaneously, they can disrupt malignant feedback loops, eliminate residual tumor cells, convert immunologically cold tumors into hot tumors, increase tumor sensitivity to adjuvant therapy, suppress recurrence, and prolong patient survival. Created in BioRender. Chen, Z. (2026) https://BioRender.com/qtd8ped.

Metal-based nanoparticles can release metal ions, such as Fe²⁺ and Cu²⁺, after degradation. These ions may enter endogenous metal ion pools or be exported through transporters such as ATP7A and ATP7B. When clearance is impaired, metal ions may accumulate and cause metal-associated neurotoxicity 101, as shown in Figure 8.

## 6. Responsive Materials for Microenvironment-Adaptive Remodeling

**Figure 9:**
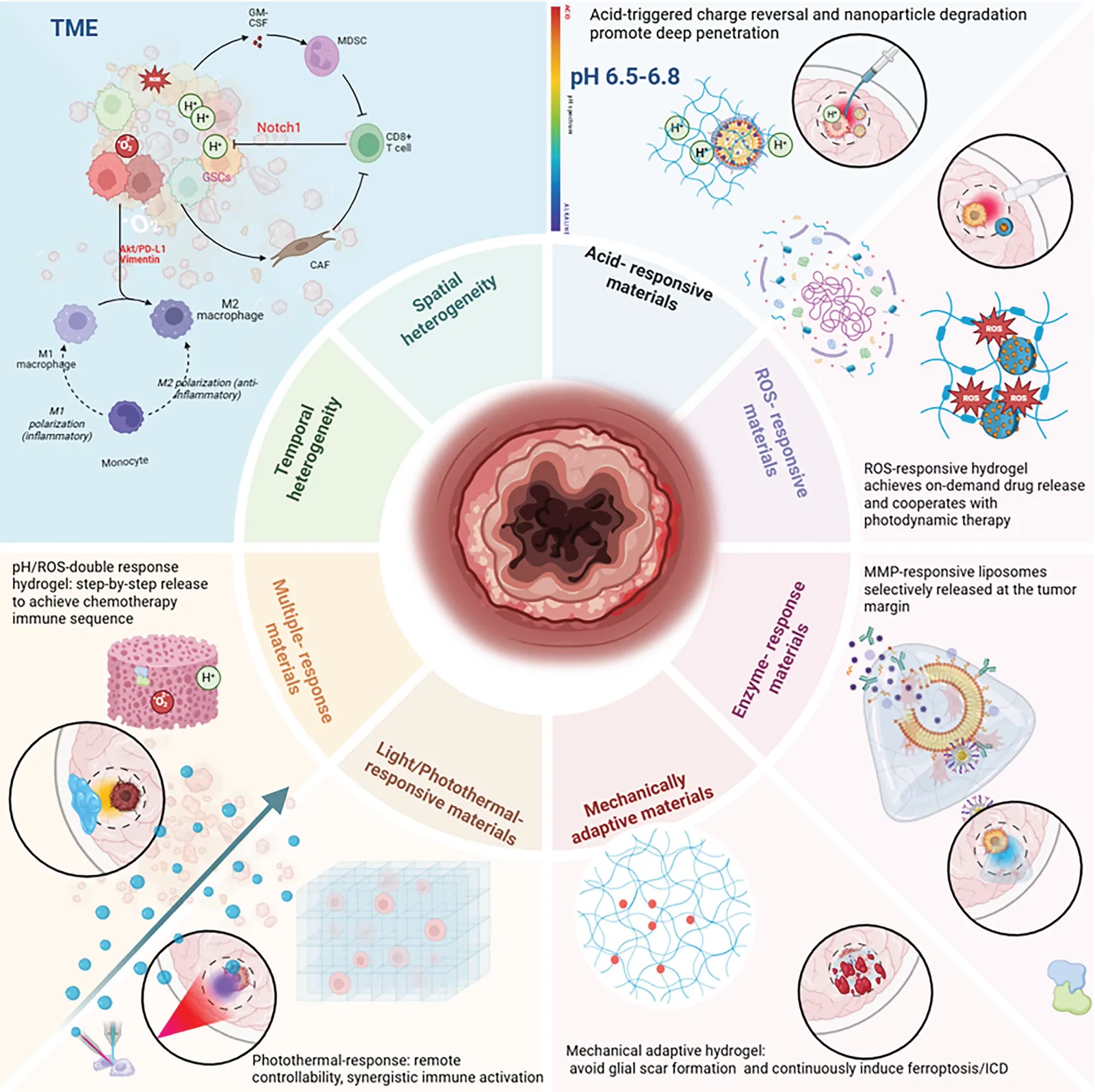
Therapeutic strategies using engineered nanomedicine to remodel the POCM and suppress GBM recurrence, including acid-responsive, ROS-responsive, enzyme-responsive, multiresponsive, and mechanically adaptive hydrogel systems. Created in BioRender. Chen, Z. (2026) https://BioRender.com/sqaa428.

Several features of the POCM can serve as release triggers, including mildly acidic pH, increased ROS and GSH, matrix metalloproteinase activity, and externally induced heating. Responsive materials use these signals to alter degradation, charge, permeability, or drug release. The following sections compare acid-, ROS-, enzyme-, multi-, photo-, thermo-and mechanically responsive designs for postoperative GBM treatment (Figure 9).

Figure 9. Therapeutic strategies using engineered nanomedicine to remodel the POCM and suppress GBM recurrence, including acid-responsive, ROS-responsive, enzyme-responsive, multiresponsive, and mechanically adaptive hydrogel systems. Created in BioRender. Chen, Z. (2026) https://BioRender.com/sqaa428.

### 6.1 Acid-responsive materials

**Figure 10:**
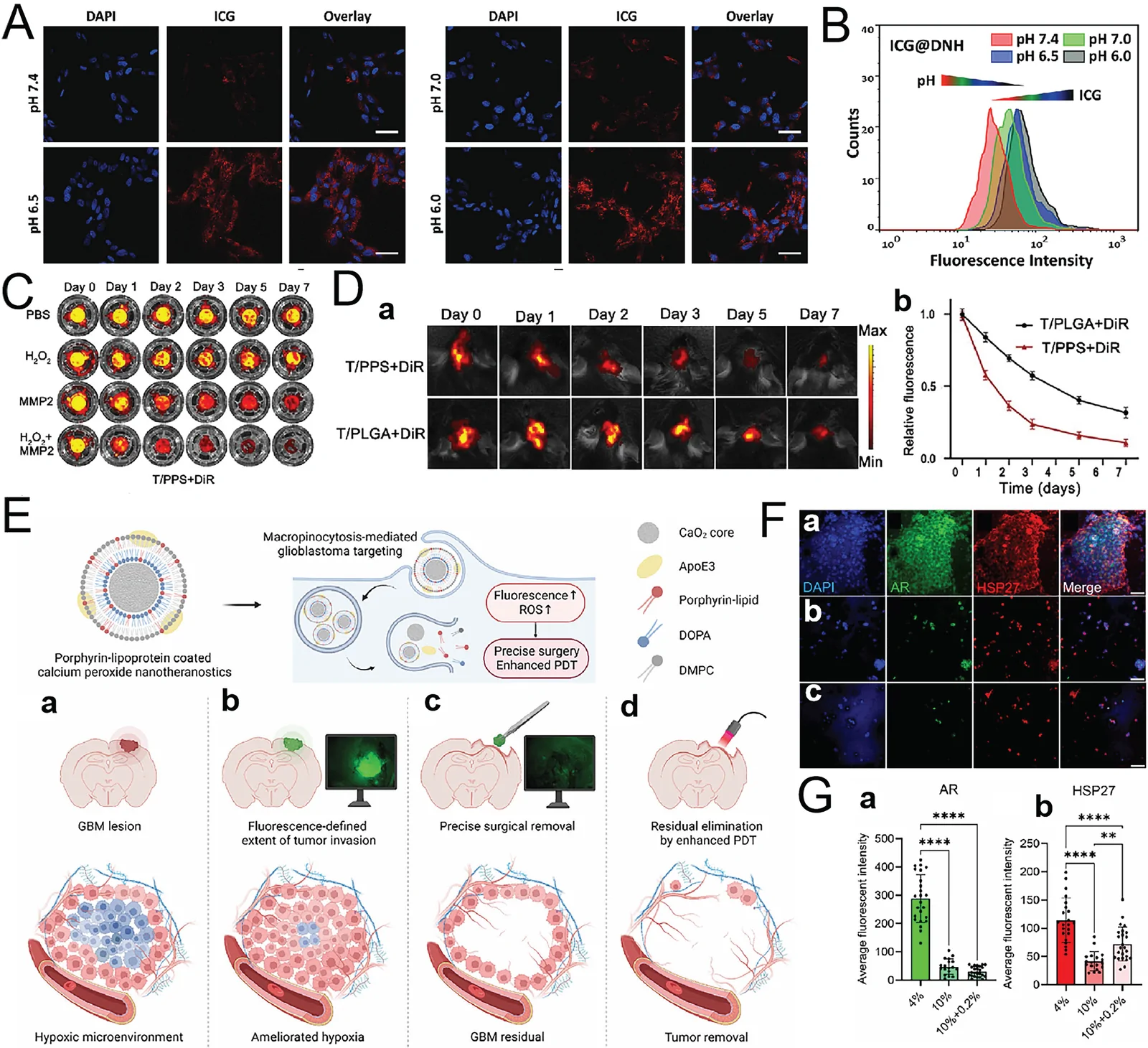
** Microenvironment-adaptive remodeling using responsive materials. (A)** ICG fluorescence images and** (B)** flow-cytometric analysis of U87MG cells after 24 h preincubation with ICG@DNH extracts under different pH conditions. Scale bar, 40 μm. Adapted with permission from Ref. 23. Copyright 2024 Wiley-VCH GmbH. **(C)** DiR release curves from T/PPS + DiR hydrogels incubated in PBS without or with MMP2 and H₂O₂. Adapted with permission from Ref. 103. Copyright 2021 the authors. **(D)** Fluorescence intensity of T/PPS + DiR and T/PLGA + DiR hydrogels at days 0, 1, 2, 3, 5, and 7 after injection into the resection cavities of GL261 tumor-bearing mice (a), together with quantitative analysis of the mouse DiR fluorescence signal at each time point (b). Data are presented as the mean ± standard error, n = 3. Adapted with permission from Ref. 103. Copyright 2021 the authors. **(E)** PLCNPs enable fluorescence-guided GBM resection and enhance postoperative photodynamic therapy through real-time tumor fluorescence imaging and alleviation of the hypoxic TME. Adapted with permission from Ref. 109. Copyright 2024 Wiley-VCH GmbH. **(F)** Immunostaining images of DI318 cells encapsulated in hydrogel models comprising 4% GelMA (a), 10% GelMA (b), and 10% GelMA + 0.2% HA (c). Adapted with permission from Ref. 112. Copyright 2025 the authors. **(G)** Mean fluorescence intensities of AR (a) and HSP27 (b) in different samples. Adapted with permission from Ref. 112. Copyright 2025 the authors.

Residual tumor cells in the POCM sustain aerobic glycolysis, reducing extracellular pH to 6.5 to 6.8, substantially below the physiological level of approximately 7.4 in normal brain tissue. This pH gradient provides an ideal trigger for the design of acid-responsive nanomaterials. Wu *et al*. 23 developed the acid-sensitive Fe³⁺/tannic acid metal-phenolic network hydrogel TP@DNH. When exposed to the acidic TME, the Fe³⁺/TA network dissociated. Released Fe³⁺ catalyzed the Fenton reaction to generate ROS, while released triptolide inhibited the NRF2/SLC7A11/GPX4 axis. This dual-pathway synergistic strategy induced ferroptosis and produced marked antitumor effects in a model of postoperative GBM recurrence (Figure 10A and 10B).

Calcium carbonate nanoparticles degrade in acidic environments and simultaneously release Ca²⁺ and their encapsulated drugs. Hou *et al*. 102 designed glioma-cell membrane-coated calcium carbonate nanoparticles loaded with DOX, termed CaDM. Under acidic TME conditions, these nanoparticles synchronously released DOX and Ca²⁺. DOX exerted cytotoxic effects and induced ICD, whereas Ca²⁺ overload caused mitochondrial injury and thrombosis within tumor vessels. Neutralization of the acidic microenvironment by calcium carbonate also downregulated cathepsin B and thereby reversed local tumor immunosuppression. Injection of CaDM mixed with the clinical hemostatic agent Surgiflo into the resection cavity prolonged median survival in tumor-bearing models from 14 d to 40 d. Wang *et al*. 36 further constructed an acid-responsive hydrogel for delivery of CD73-targeting siRNA to block adenosine-mediated immunosuppression. In this system, DOX released tumor antigens and imiquimod established a local proinflammatory micro-environment, producing a synergistic enhancement of antitumor immune clearance.

### 6.2 ROS-responsive materials

Surgical injury and metabolism of residual tumor cells substantially increase ROS levels within the POCM. ROS-responsive materials generally release drugs through cleavage of sulfur-containing or selenium-containing bonds. Zhu *et al*. 103 synthesized the composite hydrogel T/PPS + TMZ, comprising ROS-responsive poly(propylene sulfide) PPS60 and an MMP-responsive lipid, T, and loaded it with TMZ. The hydrogel formed *in situ* within the resection cavity, responded simultaneously to high local ROS and MMP levels, and continuously released TMZ, suppressing postoperative recurrence (Figure 10C and 10D). Chen *et al*. 26 encapsulated BCNU and TMZ in ROS-sensitive PLGA nanoparticles and dispersed them in a thermosensitive hydrogel. ROS stimulation at residual tumor sites caused nanoparticle degradation and chemotherapeutic release, enabling synergistic sustained treatment. A smart hydrogel, CSSH-Gel, has also recently been developed for combined sonodynamic and gas therapy. This hydrogel responds to the TME, generates ROS, releases H₂S gas, and precisely eliminates postoperative residual tumors 104.

### 6.3 Enzyme-responsive materials

There is overexpression of MMPs, mainly MMP-2 and MMP-9, in the GBM microenvironment, and they have important effects on tumor invasion and angiogenesis. Enzyme-responsive materials are mostly designed based on MMP-specific cleavable peptide sequences to realize drug release. For example, in the T/PPS hydrogel created by Zhu *et al*. 103, the incorporated glyceryl stearate lipids respond to MMPs themselves in that they can undergo hydrolysis in an MMP-rich microenvironment and thus help deliver TMZ. With regard to their dual sensitivity to both MMPs and ROS, such a dual-responsive system allows precise regulation of postoperative local chemotherapy. In the self-cascading catalytic therapy and antigen-capture scaffold developed by Yalamandala *et al*. 76, Fe²⁺ is released from an iron-containing MOF in the TME to initiate the Fenton reaction and produce ROS. Meanwhile, chloroquine contributes to increasing cytotoxicity by inhibiting autophagy. Tumor antigens released thereby bind to PU-EO-PO blocks contained in this platform, which delivers these antigens to DC cells; hence, they can present such antigens to T lymphocytes. Therefore, the level of T-cell immunity activation rises accordingly. There is also more than just responsive drug release: Barbugian *et al*. 105 describe the hyaluronic-acid-cross-linked branched MMP inhibitor hydrogel HA-MMPI; instead of responding by releasing drugs from itself, the gel fills the space left by surgical resection and selectively inhibits MMP-2 activity there, greatly decreasing cell migration in a three-dimensional culture of U87 cells.

### 6.4 Multiresponsive materials

There are many pathological factors contained in the POCM, so one kind of responsiveness cannot meet the needs of precise treatment; therefore, multi-responsive materials can realize staging and multilevel regulation of drug delivery. Chen *et al*. 26 prepared a hydrogel possessing temperature and ROS dual responsiveness. With this system, the slightly low pH value and high oxidation state inside the resection cavity could also be detected in time, thereby realizing stepwise release of chemotherapeutics. In addition, the T/PPS hydrogel created by Zhu *et al*. 103 responded to two stimuli (namely MMPs and ROS), which allowed continuous release of TMZ in the surgical site. In fact, Xue *et al*. 106 reported UMPCL, which is also a kind of sequential nanomachine regulated by a “dual-lock” logic gate. When acidity occurs, UMPCL releases Ca²⁺ and inactive peroxymonosulfate one after another. Then GSH causes Cu⁺ release; Cu⁺ can activate peroxymonosulfate and produce ROS very quickly. Through this procedure, Ca²⁺ overload occurs again, along with cuproptosis, pyroptosis, and ferroptosis; therefore, a certain period of time will elapse before these kinds of cell-death pathways take effect. This allows enough time for calreticulin display and DC maturation to occur. In the course of the above two reactions, the NIR-II fluorescence of UMPCL recovers stepwise, which provides great assistance for accurate surgical orientation. They then developed Cao *et al*. 107, who made one themselves called CP&CL@RNPPTX-Gel. It contained a built-in light emitter consisting of Ce6 linked to luminol by itself; there were also glioma-specific prodrugs of paclitaxel in nanoparticulate form, plus some copper peroxide nanodots. Therefore, they combined three types of medicine delivery: chemotherapy, CDT, and PDT, so these acted together to stop recurrence of cancer after operations had been performed.

### 6.5 Photoresponsive and thermoresponsive materials

There is also external stimulation such as near-infrared light and ultrasound. They can remotely act on materials, realize their own function, and provide spatial and temporal regulation for therapy. Nie *et al*. 108 introduced porous Pd-Cu NCs (PCNs) into the hemostatic matrix Surgiflo to obtain Surgiflo@PCN for injection into the resection cavity. When an 808-nm laser was applied, the oxidase-like, peroxidase-like, and catalase-like activities of PCNs produced ROS. At the same time, there was also a photothermal effect; therefore, they could kill possible remaining tumor cells *in situ*, induce ICD, and reverse immuno-suppression *in situ*, among other effects. They created self-disassembling porphyrin-lipoprotein-coated calcium peroxide nanoparticles called PLCNPs. Targeting occurred via macropinocytosis. PLCNPs then delivered CaO₂ inside the tumor cell and produced O₂ *in situ* to relieve hypoxia locally in the TME. Thereafter, upon laser stimulation, the photosensitizer contained in the PLCNP also generated ¹O₂. Therefore, this treatment satisfied both conditions needed if fluorescent-guided surgery was performed together with PDT afterward (Figure 10E). Then there is the work reported by Huang *et al*. 110, who invented a gelatin-based, blue-light-crosslinked, *in situ* gelling hydrogel platform. It can gel quickly *in situ* and adhere tightly to tissues, so it also lends itself well to controlled chemo-, radio-, and enhanced laser interstitial thermal therapy.

### 6.6 Mechanically adaptive materials

The physical microenvironment of the POCM substantially affects therapeutic efficacy. Jia *et al*. 91 described an injectable LFH with a mechanical strength of approximately 337 Pa, similar to that of murine brain tissue. After injection into the resection cavity, the hydrogel maintained brain water content at approximately 77% of the normal level, did not induce astrocyte activation or hypertrophy, and prevented cerebral edema and scar hyperplasia. LFH spontaneously degraded in interstitial fluid and gradually released LA and Fe³⁺. Cyclic redox reactions involving LA/DHLA and Fe³⁺/Fe²⁺ continuously generated ROS and induced ferroptosis and ICD. Additional loading with anti-PD-L1 enhanced immunotherapeutic efficacy. The study showed the importance of mechanical compatibility between postoperative implants and brain tissue for tissue protection and immune-microenvironment re-modeling. Isik *et al*. 111 created a biomimetic hydrogel platform combining brain-derived decellularized extracellular matrix (ECM) with methacrylated hyaluronic acid. The 1H3D composite had an elastic modulus of 458.30 Pa, closely reproduced the mechanical features of GBM tissue, and provided a mechanically matched *in vitro* model for POCM research. Schroyer *et al*. 112 used GelMA hydrogels to construct a structurally biomimetic three-dimensional GBM model with tunable mechanical properties, further demonstrating the key regulatory effects of ECM mechanics and composition on tumor-cell behavior (Figure 10F and 10G). Recent studies have shown that matrix stiffness regulates GBM migration and chemoradiotherapy responses through chromatin condensation, indicating that postoperative material design should fully account for the viscoelasticity of brain tissue 113.

Responsive materials are most useful when the trigger is present at the right concentration and time in the human cavity. More complex designs now combine several chemical inputs and, in some cases, mechanical adaptation. Further development should focus on staged responses that can be verified experimentally, integration with immunotherapy where ICD is intended, and material compositions that can be cleared safely after treatment.

### 6.7 Safety considerations in responsive-material design

Safety should be treated as a design variable rather than as a final validation step. Peptide, PLGA, and lipid materials can yield amino acids, lactic acid, or fatty acids after degradation 114-116, whereas inorganic ferroptosis- or cuproptosis-inducing particles may require an active clearance strategy, such as enhancement of microglial extracellular-vesicle release through ERK1/2 85. Low-swelling formulations are preferable in the postoperative brain because edema or compression can have serious consequences. Mechanical properties should also remain close to those of brain tissue; an elastic modulus in the approximate range of 0.1 to 1 kPa has been proposed to limit chronic glial activation 117.

## 7. Patient-Specific Modeling and Personalized Remodeling Strategies

Tumor heterogeneity largely explains why a treatment showing efficacy in standard GBM models often fails in individual patients. Even tumors with the same histological grade can exhibit substantial differences in genotype, cellular composition, and treatment response 118,119; moreover, distinct regions within a single tumor may behave quite differently. Conventional cell lines and most animal models reflect only a narrow slice of this diversity. In contrast, three-dimensional bioprinting 120 and microfluidic platforms 121 now enable postoperative drug formulations to be tested in patient-derived tissue constructs that preserve a more physiologically relevant extracellular environment.

### 7.1 Three-dimensional bioprinting of resection cavity models

**Figure 11:**
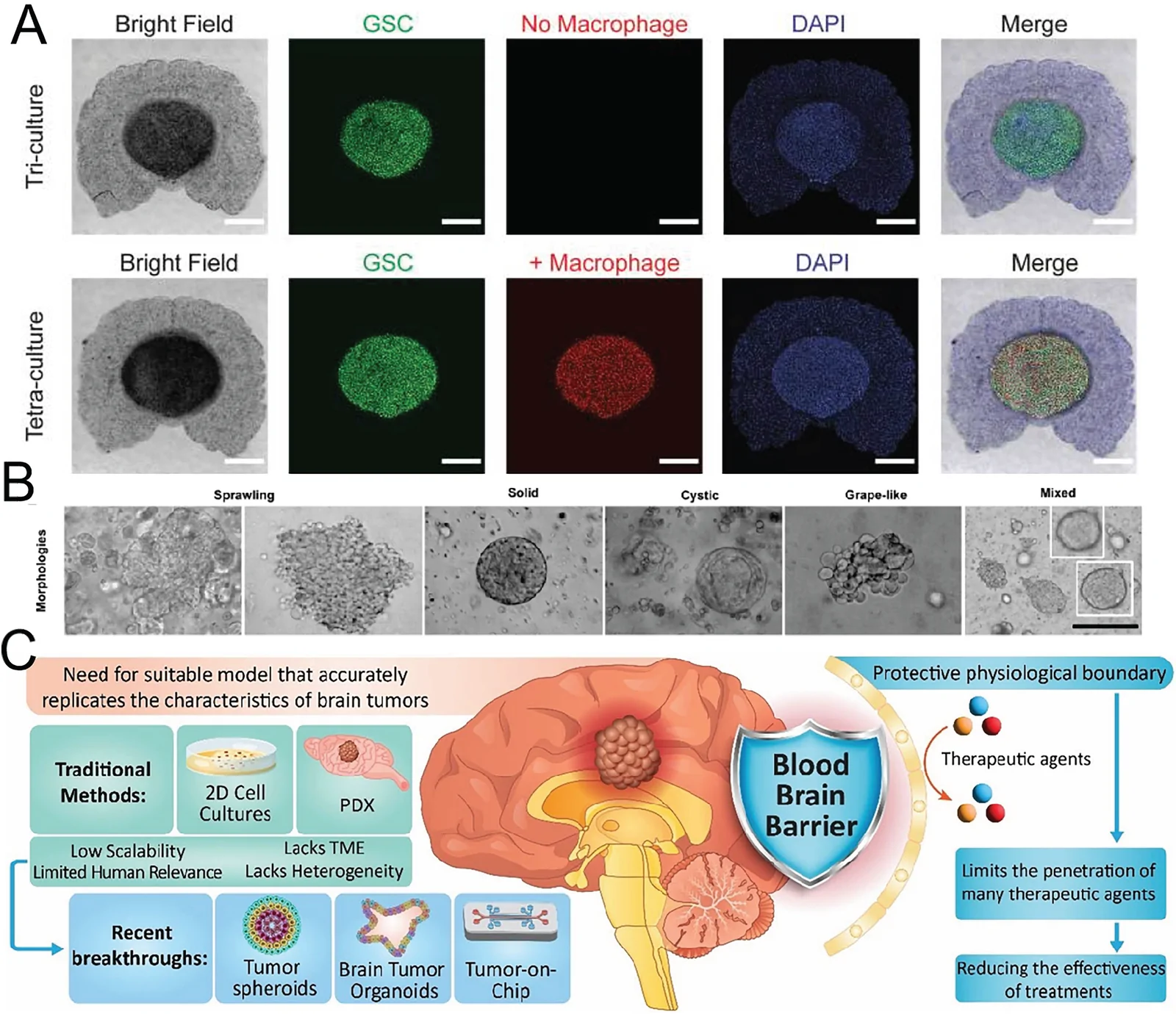
** Patient-specific modeling and personalized remodeling strategies. (A)** Bright-field and immunofluorescence images of three-dimensional glioblastoma models in tri-culture and tetra-culture. GSCs are labeled with green fluorescent protein and macrophages with mCherry. Adapted with permission from Ref. 123. Copyright 2020 Chinese Academy of Sciences. **(B)** Representative bright-field images of different morphologies in the GBM-3DP model. Adapted with permission from Ref. 124. Copyright 2026 Elsevier B.V. **(C)** Different methods for constructing brain-tumor models, including organoids, three-dimensional bioprinting, and microfluidic chips. Adapted with permission from Ref. 128. Copyright 2025 the authors.

Standard 2D monolayer cultures fall short of mirroring the native 3D tumor architecture and cell–ECM crosstalk. Consequently, drug-sensitivity readouts from these systems often deviate considerably from actual *in vivo* responses 120. Three-dimensional culture technologies, especially rapidly developing three-dimensional bioprinting, can generate more physiologically representative tumor models by accurately reproducing key physicochemical signals involved in cell-cell and cell-ECM interactions 122,123. Schroyer *et al*. 112 used GelMA hydrogels to establish a structurally biomimetic three-dimensional GBM model with tunable mechanical properties. Embedded bioprinting was used to create channels within the hydrogel scaffold and reproduce GBM ECM features. GBM cells showed greater functional activity in softer matrices, and addition of hyaluronic acid further improved the ability of the GelMA matrix to mimic the native GBM TME. Building on this work, Tang *et al*. 123 used three-dimensional bioprinting to construct a three-component TME model comprising GBM stem cells, astrocytes, and macrophages. The model revealed intercellular dependencies and provided a technical basis for reproducing interactions between tumor and immune cells within the resection cavity (Figure 11A).

Establishing three-dimensional models from primary cells isolated from a patient's surgical tissue is a prerequisite for personalized treatment evaluation. Li *et al*. 124 used digital light processing (DLP) three-dimensional bioprinting to establish the first GBM model capable of serial passage and selected a chemically defined ECM-mimetic hydrogel with physicochemical properties matching patient-derived GBM tissue (Figure 11B). The platform achieved three major advances. First, it supported continuous culture of primary GBM for more than 6 weeks and stable passage for at least five generations. Second, it preserved the histopathological architecture, cellular diversity, and somatic mutation profile of the original tumor during long-term culture. Third, evaluation with CAR-T-cell therapy demonstrated that passaged bioprinted GBM models could predict responses to immunotherapy.

The efficiency and throughput of drug screening are major factors limiting the clinical implementation of personalized treatment evaluation. Tripathy *et al*. 125 created a high-throughput three-dimensional culture platform based on modular star-shaped PEG-glycosaminoglycan hydrogels. The platform enabled independent control of key ECM physicochemical parameters in 384-well plates and three-dimensional encapsulation of patient-derived GBM cells. Transcriptomic analysis showed that tumor programs from both primary and recurrent GBM were successfully reproduced under three-dimensional culture conditions. Subsequent high-throughput drug screening using multiregional patient-derived glioma organoids demonstrated that drug responses differed not only among patients and tumor types but also among regions of the same tumor, indicating that spatial heterogeneity critically influences treatment sensitivity. The GliaMimic platform established by Camenisch *et al*. 126 applied the standard Stupp regimen to patient-derived GBM organoids and longitudinally assessed treatment response and recurrence using multiparametric dynamic monitoring.

For postoperative treatment, the principal value of three-dimensional bioprinted models is their ability to use specimens obtained intraoperatively to rapidly establish a model and evaluate multiple nanomedicines and combination regimens within a short period 120,123,127. Current models, however, remain limited in their reproduction of resection cavity-specific pathology. First, reconstruction of the POCM remains insufficient. Most models include only tumor cells and matrix components and do not reproduce immune-cell infiltration and functional changes caused by surgical injury. Second, mechanical simulation is inaccurate because the mechanical boundary conditions of the resection cavity differ fundamentally from those of intact tumor tissue. Third, integration of vascular and immune components remains incomplete. Incorporating perfusable microvascular networks and immune-cell systems is still a major technical challenge in constructing a complete biomimetic TME.

### 7.2 Microfluidic chips for drug screening

Through microscale fluid manipulation, microfluidic chips can achieve multidimensional biomimetic reconstruction of the TME and have substantial potential in brain-tumor diagnosis and treatment 121,128. Its advantages are evident in several aspects. Three-dimensional microfluidic structures can recreate a biomimetic tumor microenvironment. Compared with conventional methods, they increase the capture efficiency of exosomes and circulating DNA by three- to fivefold. The microchannel design increases the blood dilution ratio from 10² in conventional assays to 10⁶, overcoming the limitations imposed by low biomarker concentrations. The integrated detection module also enables full-process automation from sample capture to result output.

Hariri *et al*. 128 reviewed recent advances in brain tumor organoid research, with a focus on vascularization strategies and their effects on model fidelity. They also evaluated the potential applications of organoid platforms in drug screening and personalized therapy, providing a systematic framework for the future development of this field (Figure 11C).

Tumoroid-On-a-Plate is a novel open-surface microfluidic platform that integrates tumor aggregates, stromal cells, and ECM components to reproduce the microenvironments of GBM and pancreatic ductal adenocarcinoma with relatively high fidelity 129. The platform has been used to screen TMZ and iron chelators as single agents and in combination and to evaluate their toxic and proapoptotic effects on tumor-like structures within a complex ECM. Another study constructed a three-dimensional GBM model integrating human TME cells, ECM-mimetic materials, and a microfluidic platform. Tumor spheroids comprised primary GBM cells, GBM-8 cancer-associated stem cells, microglia, and astrocytes and were encapsulated in hydrogels of different stiffnesses to reproduce the role of the ECM in tumor progression. This model was used to evaluate the effects of bortezomib-loaded nanoparticles on spheroid invasion 130. Manoharan *et al*. 131 assembled a three-dimensional microfluidic tumor-on-a-chip model that successfully reproduced the perivascular GBM niche and used monocyte membrane-coated nanoparticles to target drugs to abnormal tumor microvessels.

Microfluidic chips can also monitor dynamic responses during GBM treatment in real time. Youngblood *et al*. 132 developed a microfluidic device that captured biomarkers released by tumor cells to predict patient responses to chemotherapy. The GlioME method proposed by Zheng *et al*. 133 generated glioma organoids from patient-derived tissue while retaining microenvironmental information. The organoids preserved the genetic and epigenetic features of the original tumor as well as cellular interactions within the TME and accurately predicted patient responses to the MET inhibitor vebreltinib. Combining microfluidic technology with evaluation of CAR-T-cell efficacy has also become an important research direction. Zhu *et al*. 134 established a combined microgravity-microfluidic platform that integrated GBM organoids cultured under microgravity with microfluidics, providing a more physiologically relevant environment for evaluating CAR-γδ T-cell efficacy.

From a postoperative perspective, microfluidic chips can use patient tumor tissue to construct individualized models within several days after resection and evaluate multiple nanomedicines, combination regimens, and dosing sequences, providing experimental support for clinical decision-making. Incorporating computational fluid dynamics into an on-chip CFD design framework may further improve the engineering precision and biological fidelity of microfluidic design 135.

## 8. Current Status, Challenges, and Future Directions of Clinical Translation

Preclinically, considerable evidence supports the efficacy of engineered nanogels in preventing the recurrence of gliomas after surgery. This conclusion holds true whether one considers results based on cell cultures alone or data from experiments performed in rodents 136. Nevertheless, considerable problems still need to be solved before such encouraging news can benefit patients directly. Many difficulties remain before bench discoveries are brought to bedside use. There are several reasons why this transition has been slow: first, there appears to be a considerable drop-off in effectiveness once clinical phases begin. Second, existing animal models cannot predict what will happen when these compounds are administered to people. Third, scaling up reactions presents considerable difficulty. Fourth, little regulation exists. Finally, two inherent limitations prevent progress. On one hand, gliomas exhibit great diversity in the kinds of molecules they contain. On the other, much remains unknown about their related immunity 137-139.

### 8.1 Ongoing clinical trials: from BCNU wafers to next-generation nanogel systems

In fact, the idea behind locally delivering chemotherapy immediately after removal of GBM arose long ago. There was no lack of precedent: the Gliadel® Wafer (BCNU) had been on the market almost thirty years earlier. According to the most important study ever carried out involving a placebo group as the control, median survival reached 31 weeks, compared to merely 23 weeks in the control. This result brought about FDA approval first for recurrent GBM in 1996 140 and then for primary GBM in 2003 141, but with wider application came a raft of problems. There was little room for spreading out, depletion occurred rapidly because of burst release, there was also resistance to BCNU itself, CSF leakage developed, and intracranial pressure increased. As these difficulties took their toll one by one, they erased whatever benefit remained regarding overall survival 139, 142. Hence, researchers began looking elsewhere, turning instead to something far more advanced: nanogelled compounds. Some examples even entered the clinic soon afterward. A phase-wise account appears below in Table 3.

#### (1) GammaTile, an implantable device for immediate postoperative radiotherapy in GBM

GammaTile, developed by GT Medical Technologies, is a US Food and Drug Administration-approved bioabsorbable collagen-matrix device embedded with radioactive Cs-131 seeds. The device is designed for direct implantation into the resection cavity during tumor removal, allowing local radiotherapy to begin on the day of surgery. In January 2026, the first patient with newly diagnosed GBM was enrolled in the US multicenter randomized controlled BRIDGES trial (NCT06462443), which is expected to provide higher-level evidence for this immediate postoperative radiotherapy strategy.

#### (2) Registration-oriented phase II trial of the irinotecan sustained-release implant Irinotecan-ChemoSeed

Irinotecan ChemoSeed, developed by CRISM Therapeutics, is a biodegradable implantable technology platform that delivers irinotecan directly to the tumor resection margin in a sustained-release form for postoperative local chemotherapy. In September 2025, CRISM announced that its open-label phase II safety and efficacy trial had received approval from the UK Medicines and Healthcare products Regulatory Agency and ethics committee authorization, with formal initiation planned for the first quarter of 2026. The study was designed as a registration-oriented trial intended to directly support a subsequent marketing authorization application.

#### (3) Cerebraca® Wafer, a locally implanted formulation targeting the RTK network

Cerebraca® Wafer, developed by Taiwan-based Everfront Biotech, has completed a phase I/IIa clinical trial. Median survival was 15.7 months in the overall study population and increased to 26.2 months in the subgroup with high RTK expression. Notably, no drug-related grade 3 or higher serious adverse events were observed. Brain-tissue diffusion experiments showed that the active ingredient diffused more than 2 cm in canine brain tissue while maintaining a high local concentration and very low systemic exposure.

#### (4) Phase I progress of CLD-101, neural stem cells carrying an oncolytic virus

CLD-101, developed by Calidi Biotherapeutics, consists of neural stem cells carrying an oncolytic virus and is delivered directly into the brain after surgery. A phase I clinical-trial update reported that 14 patients had received treatment. All participants tolerated the therapy well, and no unacceptable safety signals were identified.

#### (5) GLIORA, a long-acting thermosensitive hydrogel from Nanoform and Revio Therapeutics

In October 2025, Nanoform Finland announced a strategic collaboration with Revio Therapeutics to jointly develop and commercialize GLIORA. The product is a nanomedicine-composite hydrogel containing olaparib and TMZ and is intended for local delivery to the POCM. Using its proprietary CESS® technology and hydrogel platform, Nanoform has prepared a nanomedicine prototype under GMP conditions at its Helsinki manufacturing facility. Preclinical development has entered the late stage, and a phase I clinical trial is expected to begin in the second half of 2026. If results are favorable, commercialization is planned for 2029 to 2030.

In addition to the programs that have entered clinical development, several nanogel systems have shown notable translational potential in preclinical studies. Shang *et al*. 20 documented a hydrogel based on a STING agonist prodrug, Wang *et al*. 36 developed a lymph-node-inspired hydrogel responsive to acidic conditions, and Nie *et al*. 108 assembled a nanozyme hydrogel combining photothermal effects with immunomodulation. All three produced favorable therapeutic results in animal experiments. Bastiancich *et al*. 143 also developed a GemC12-loaded lipid nanocapsule hydrogel for postoperative local chemotherapy of GBM. This system may be particularly valuable during postoperative management before standard chemoradiotherapy begins.

#### (6) Clinical translation challenges for GBM immunotherapy and nanomedicine

For decades, hundreds of preclinical studies have established that many different immunotherapeutic regimens possess antitumor activity in mouse models of brain cancer. However, for years there has been failure of clinical translation of GBM immunotherapy and nanomedicine. A systematic review and meta-analysis of 12 randomized controlled trials (RCTs) demonstrated that in general the overall survival of patients treated with immunotherapy was less than in those patients who received no treatment or alternative therapy. These 12 studies together analyzed the results of the overall survival of 2487 patients in the 12 RCTs of GBM immunotherapy. The resulting pooled hazard ratio for overall survival was 1.16 (95% CI = 1.06–1.28) indicating that in general the overall survival of patients treated with immunotherapy was less than in those patients who received no treatment or alternative therapy. Importantly, no single study provided evidence of improved overall survival of patients with GBM who were treated with immunotherapy when compared with patients with GBM who were treated with no treatment or with alternative therapy. As a consequence of these failed attempts at clinical translation of preclinical studies of GBM immunotherapy, hundreds of preclinical studies of GBM immunotherapy continue to be performed every year and the results of almost all of these hundreds of studies are never published and, therefore, never bring about any change in clinical practice. In addition, and of greater significance, many recently completed phase III clinical trials of different immunotherapies for the treatment of patients with newly diagnosed GBM have failed to provide evidence of improved overall survival. For example, the CheckMate 14 study was a first phase III study to compare nivolumab with bevacizumab as a treatment for patients with recurrent GBM. This study did not meet its primary endpoint of improved overall survival 145. More recently, the results of CheckMate 498 and CheckMate 548 were published. CheckMate 498 was a study of nivolumab in combination with radiotherapy for patients with newly diagnosed unmethylated MGMT promoter GBM. This study did not meet its primary endpoint of overall survival and, therefore, provided no evidence of clinical benefit of nivolumab for patients with this subset of GBM 146. The CheckMate 548 study was a study of the addition of nivolumab to the current standard of care of chemoradiotherapy for patients with GBM that have an unmethylated MGMT promoter. This study also did not provide evidence of improved overall survival with the addition of nivolumab to the current standard of care of chemoradiotherapy for patients with GBM that have an unmethylated MGMT promoter. Importantly, however, CheckMate 548 did provide evidence of exploratory endpoints. Therefore, CheckMate 548 did retain the possibility of approval for regulatory purposes for the treatment of patients with GBM that have an unmethylated MGMT promoter. However, as already noted, five of the six recently published phase III immunotherapy trials for the treatment of GBM provided no evidence of clinical benefit for any of the endpoints that were used to evaluate the results of the studies and, therefore, provided no evidence to support approval for regulatory purposes for the treatment of patients with GBM of the treatments that were tested in the studies. Thus, of recently completed phase III studies of GBM immunotherapy, only one study, CheckMate 548, provided evidence of clinical benefit of any of the endpoints that were used to evaluate the results of the studies and, therefore, provided evidence to support approval for regulatory purposes for the treatment of patients with GBM of the treatments that were tested in the studies. Monotherapy with the two single agents CTLA-4 inhibitors ipilimumab and tremelimumab also failed to provide proof of concept for immunotherapy of patients with GBM. In a systematic review of 106 clinical trials of cancer immunotherapies, no improvement in overall survival over standard of care was reported for any of the many different approaches to cancer immunotherapy 148.

There are several factors contributing to the problems of translation of preclinical studies to clinical setting. First, GBM has low tumor mutational burden compared to cancers that respond well to immunotherapy, such as melanoma and non-small cell lung cancer. Thus, GBM has limited number of tumor-specific antigens that can be recognized by T cells for immunotherapy. However, even in the presence of a limited number of antigens, there are several factors that prevent successful priming of T cells for immunotherapy. Most importantly, GBM contains strongly immunosuppressive tumor micro-environment, where glioma-associated macrophages, microglia and myeloid-derived suppressor cells represent the dominant cell types that secret IL-10 and TGF-β to prevent entry of cytotoxic T cells into tumor tissue. In addition, there are strong interpatient and intratumoral heterogeneities that lead to continuous change in expression of antigens recognized by T cells for killing of tumor cells, resulting in antigen loss and subsequent immune escape of remaining tumor cells in treated patients. Finally, there are several physical barriers to penetration of therapeutic antibodies and effector cells into GBM. First, blood-brain barrier in brain contains strongly barrier-like structure, called blood-tumor barrier in GBM. This physical barrier prevents entrance of most therapeutic antibodies into GBM for their desired actions. Recently, a single-cell spatial-transcriptomic analysis of human GBM following neoadjuvant treatment with nivolumab, a PD-1 inhibitor, showed that neither tumor cells nor tumor-associated macrophages displayed significant changes in gene-expression profiles in response to treatment with PD-1 inhibitor. These findings provided direct evidence for lack of effective translation of nivolumab treatment into changes in GBM 150.

Nanomedicine has achieved considerable preclinical success but faces similar translational difficulties. Intravenously administered nano-therapeutics must first cross the BBB, then pass through complex tumor tissue, and finally penetrate the dense extracellular matrix before reaching target cells. Nanomedicines with relatively simple functions have shown limited efficacy in clinical trials 151. Even when early pharmacokinetic data are favorable, large-scale manufacturing, regulatory requirements, and potential immunogenicity remain major barriers. Nanomedicines have also been reported to cause severe adverse effects. These include rapid immune recognition of nanocarriers and accelerated clearance from the bloodstream. Early efficacy signals have repeatedly failed to produce survival benefits, indicating a systematic failure of clinical translation 144.

### 8.2 The challenge of clinical translation

Material performance is only one part of translation. Three less-discussed constraints may determine whether a local treatment works in patients: the actual physical contents and shape of the resection cavity, the limited distance that a released agent can travel through brain tissue, and the uncertain predictive value of large-animal models.

#### 8.2.1 The physical resection cavity environment

Most current preclinical studies evaluate hydrogel performance in clean and blood-free simulated resection cavities in mice or rats 152,153. These models differ greatly from actual surgical conditions in humans. The postoperative resection cavity in patients with glioma is a highly dynamic and compositionally complex space. Persistent bleeding of varying severity is common and may be accompanied by hematoma formation or cerebrospinal fluid leakage. Blood components such as thrombin, fibrinogen, and erythrocyte fragments can interfere with the gelation of hydrogel precursor solutions 154,155. In ionically or enzymatically crosslinked hydrogels, these components may occupy crosslinking sites or alter the local pH. This can result in incomplete gelation or a marked reduction in mechanical strength 156. In thermosensitive hydrogels such as Pluronic and PEG-PLGA-PEG, dilution by blood may shift the phase transition temperature and prevent rapid gelation at body temperature 157. Gelation failure may cause drug loss through cerebrospinal fluid circulation and may even induce chemical meningitis.

The surgical resection cavity is often highly irregular in shape. It may extend along white matter tracts in a tunnel-like pattern and leave unfilled spaces. Conventional hydrogel delivery usually relies on a single-point injection and cannot achieve uniform distribution throughout a complex cavity. After gelation, the hydrogel must withstand continuous shear forces caused by brain pulsation and cerebrospinal fluid flow. It must also avoid excessive swelling that could compress adjacent brain tissue 158.

Cerebral edema is another important concern. Resection of malignant gliomas can cause varying degrees of cerebral edema 159. Excessive swelling of implanted hydrogels or hydrogel-induced local inflammation may aggravate postoperative edema. This can lead to neurological damage and increased intracranial pressure. To address this issue, Jia *et al*. 87 described an injectable lipoic acid-iron hydrogel known as LFH. Its mechanical properties matched those of brain tissue, with a modulus of 337 ± 8.06 Pa. After injection into the resection cavity, LFH maintained brain water content at approximately 77%, which was within the normal range. It did not induce astrocyte activation or hypertrophy and prevented cerebral edema and scar formation. This study shows that mechanical matching between the hydrogel and host brain tissue is essential for preventing edema and chronic inflammation. GliaTrap, which is composed of hyaluronic acid and type II collagen, also uses a non-swelling biomimetic design. It has been shown not to induce brain inflammation or local swelling 160. Overall, non-swelling or low-swelling properties and mechanical parameters that match brain tissue are basic requirements for safe intracranial implantation.

At the level of nanomaterial diffusion, the postoperative intracranial environment is much more complex than intact tissue models. Hematomas, necrotic tissue debris, and extensive inflammatory cell infiltration in the surgical region can alter the effective diffusion coefficient of nanomedicines 161. Glycosaminoglycans and proteoglycans are abundant in the brain extracellular matrix. They form both physical and chemical barriers that substantially restrict nanoparticle movement. Studies have shown that nanoparticles no larger than 100 nm and densely coated with PEG diffuse more rapidly and cover a larger area in the brain parenchyma. In contrast, larger or positively charged particles are severely restricted. However, these conclusions are mainly based on healthy brain tissue or intact tumor tissue. Systematic experimental data on the actual diffusion of nanomaterials within postoperative resection cavities containing hematomas and inflammation remain lacking. This issue requires dedicated investigation.

#### 8.2.2 Physical limits of drug diffusion

Local recurrences in GBM patients often arise near the surgical margin where infiltrating tumor cells travel in white matter tracts over distances of up to 2 to 4 cm from the surgical cavity. In contrast, the effective diffusion distance of most nanomedicines is of the order of 5 mm and thus does not extend to cover the entire tumor cavity, as would be required for a simple local depot to be effective for GBM. Passive diffusion is hampered by the high cell density within the brain and by components of the extracellular matrix, which strongly impede the diffusion of larger molecular weight species and particles.

Convection-enhanced delivery of micromole-cules can result in the distribution of therapeutic entities over centimeter-scale distances using the convection of a fluid under pressure gradient. Local delivery using an implanted catheter, however, is not ideal for uniform distribution throughout the brain and thus does not provide sufficient tumor coverage to treat all the tumor cells. Hence, for diffuse, infiltrating residual tumor cells distant from the site of local delivery, systemic distribution of chemotherapeutic agents or immunotherapeutic reagents is required to achieve a cure of all tumor cells in the brain.

#### 8.2.3 False hope from large-animal models

In order to assess the ability of nanogel systems to regulate the amounts of T cells and macrophages that are typically part of the immune system (in addition to being part of the TME) that need to be remodeled by POCM therapy in human brains, large animal models with intact immune systems cannot be used because the amount of human brain tissue that can be implanted in these animals is typically too small.

However, the spontaneous canine glioma model, which has an intact immune system and is characterized by a species-specific TME, is currently considered to be the most predictive model for POCM therapy 168, 169. Although the cost of each dog can be in the tens of thousands of dollars and recruitment can take long, the number of dogs that can be studied in a given time frame is limited. Each dog is unique with regards to genetics and therefore most studies are based on a small number of animals (5–10) which severely limits the statistical power and reproducibility of the data. None of the prospective studies have been able to demonstrate that successful local hydrogel treatment of spontaneous canine gliomas translates into similar successful treatment in human clinical trials. The safety and pharmacokinetic studies in canine models are of primary value.

Rather than hoping for one magic model to answer all questions in the preclinical screen for POCM, a validation-by-tiers approach would be far more rational. First, use immunocompetent syngeneic or transgenic mice to examine the novel mechanistic points and initial efficacy of a new POCM nanogel formulation. Then, for POCM-mediated immune remodeling in the postoperative period, use the surgical rodent model. In between, for issues of pharmacokinetics, device-handling, procedural-safety, etc., move to non-human primates. Finally, for safety assessment and for other key translational issues, utilize a small number of studies in spontaneous canine glioma, keeping in mind that such studies are very, very expensive and highly, heterogeneously variable.

#### 8.2.4 Risk of excessive local immune activation and the efficacy-toxicity window

Local delivery of immunomodulatory agents avoids systemic toxicity but may trigger immune overactivation within the confined intracranial space 170. The brain has long been considered an immune-privileged organ. Excessive immune responses in this region can cause peritumoral edema, seizures, autoimmune encephalitis, and even catastrophic cytokine release syndrome 171.

The STING pathway is a key target in POCM-based immunotherapy and a representative example of this problem. STING activation induces type I interferon responses and promotes antitumor immunity. However, aberrantly sustained STING signaling can also cause detrimental neuroin-flammation and neurodegenerative changes 172. In microglia and peripheral immune cells, STING-dependent signaling regulates early inflammatory responses and significantly affects outcomes after brain injury 173. Intracranial delivery of STING agonists therefore requires precise spatiotemporal control to prevent off-target neuroinflammation. Nanomedicines and sustained-release hydrogels have distinctive potential in this regard, but the problem has thus far been resolved only to a limited extent.

Similarly, TLR agonists released at supra-physiological concentrations can induce abundant production of proinflammatory cytokines such as TNF-α, IL-6, and IL-1β 174. Studies have shown that intracerebral injection of potent TLR agonists such as LPS aggravates neuroinflammation, increases TNF-α levels, and promotes infiltration of CD11b⁺ immune cells into the brain parenchyma 175. Smart biomaterials can selectively activate immune cells within tumor tissue while limiting excessive spread of systemic immune responses, thereby partially mitigating these risks.

Local immune activation is likely to have a bell-shaped dose-response curve. Too little activation leaves the suppressive niche intact, whereas too much can injure normal brain. Each immunomodulator therefore needs a defined exposure range, and the release profile should keep local concentrations within it. Biomarkers of inflammation may help, but feedback-controlled materials will require validation against neurological toxicity as well as tumor response.

### 8.3 Scale-up bottlenecks in formulation processing and GMP manufacturing

Scaling a gel from a laboratory batch to a GMP product is not a simple increase in volume. Thermosensitive hydrogels and nanogel composites contain several interacting components, and small changes in polymer structure, particle-size distribution, or gelation kinetics can alter performance 176. These variables need to be defined as critical quality attributes before manufacturing is expanded.

#### (1) Polymer chemical synthesis and quality control

Thermosensitive hydrogels are functionalized by polymer chains with certain characteristics, such as the molecular weight distribution, the block-composition and the terminal groups. Anwar *et al*. 177 set up a semicontinuous production line for PLGA/PEG-PLGA-microspheres, which is equipped with a mixing-emulsification-module and a real-time-monitoring-module. A translation of the polymer synthesis to preclinical studies for a mPEG-PAla-based thermosensitive hydrogel was conducted by Wang *et al*. 178 The resulting polymer powder could be sterilized by UV-irradiation.

#### (2) Integration of nanoparticles with the gel matrix

Challenges in scaling up nanogel-based composite systems include uniform distribution and stabilization of nanoparticles within a hydrogel matrix. The nanoparticles need to maintain uniform particle size, surface charge, and drug loading during scaling up of the nanogel-based composite system. The GLIORA program, a joint program between Nanoform and Revio Therapeutics (now part of Revio Therapeutics – a Gossamer Bio company), is an example of an industrial program that integrates production of nanomedicine with GMP-manufactured hydrogels. At its Helsinki GMP facility, Nanoform directly prepares nanomedicines using its proprietary CESS® supercritical-fluid technology and embeds them in a thermosensitive hydrogel system. Application of quality by design (QbD) principles is particularly important when evaluating the stability of such composite formulations. Dalwadi *et al*. 179 applied QbD to thermosensitive hydrogel development and defined the quality target product profile and critical quality attributes. Troiano *et al*. 180 successfully extended QbD principles to industrial scale-up of polymeric targeted delivery platforms.

#### (3) Sterilization and terminal sterilization processes

Sterility assurance requirements are stringent for intracerebral implants and injectable formulations, yet gel formulations are often sensitive to conventional terminal sterilization methods such as heating and irradiation. De Lauretis *et al*. 181 compared the effects of moist heat, dry heat, gamma irradiation, and electron-beam irradiation on the functional properties of 30% w/v Poloxamer 407 hydrogel. Electron-beam irradiation at 15 to 25 kGy preserved hydrogel elasticity, gelation, and structural properties while increasing mechanical tolerance and slowing swelling, making it the most suitable sterilization method for this thermosensitive hydrogel system. In preclinical translation of a thermosensitive mPEG-PAla hydrogel, Wang *et al*. 178 further found that ultraviolet irradiation efficiently sterilized the polymer powder, its aqueous solution could be rapidly prepared within 15 min, and the storage stability of a prefilled-syringe formulation exceeded 6 months.

## 9. Outlook

Some local nanogel concepts are approaching clinical feasibility, but ROS-responsive systems, enzyme-responsive hydrogels, and local cell-therapy platforms remain largely preclinical. Academic studies often prioritize mechanism and short-term efficacy. Manufacturing groups must also consider batch consistency, storage, sterilization, cost, and a regulatory pathway. The mismatch between these priorities contributes to the loss of candidates during translation. Reviews by Wang *et al*. 182 and Riccobelli *et al*. 183 identify scale-up, process standardization, and regulatory requirements as recurring barriers for GBM nanodelivery systems.

Closer academic-industry collaboration is particularly valuable when it begins before the formulation is fixed. Chemistry, manufacturing, and controls (CMC), stability, and manufacturability should be assessed during preclinical development, not after efficacy studies are complete. Early discussion with regulatory agencies through pre-investigational new drug meetings can also clarify the nonclinical and CMC evidence expected for a first-in-human study 176,184.

Large-animal work presents a different problem: studies are costly, difficult to standardize, and subject to strict ethical review. The US National Institutes of Health-funded canine immunotherapy consortium U01CA224151 has established a consortium of researchers to perform studies of combination of therapies for spontaneous malignant glioma in dogs with the goal of harmonizing the critical elements of a complete study, i.e. surgery, implant assessment, imaging, and histopathology. Other approaches include losartan and propranolol in conjunction with a tumor stem-cell vaccine that produced objective responses in 20% of dogs with glioma 169. Xenografts of porcine-human glioma have been used to validate intraoperative image updating for brain-shift correction 165. These models can be very useful to test issues related to procedural feasibility, pharmacokinetics, and safety; however, they are very poor at predicting efficacy in humans.

Translation of the engineered material to a reproducible medical product represents a significant change from a successful laboratory formulation. Next generation postoperative local treatment for use in the GBM recurrence after surgery setting includes development of Irinotecan ChemoSeed, Cerebraca® Wafer, and GLIORA. These nanomedicines will require to be manufactured under GMP regulations and show sufficient preclinical and clinical data to demonstrate safety and efficacy for use in a human cavity. Work is required among material scientists, neuro-oncologists, surgeons, manufacturers, and regulators.

## 10. Conclusion

Surgery for brain tumors such as GBM typically leaves behind a significant amount of tissue in the resected cavity. This residual tissue can interact with the altered tissue surrounding the resection site, leading to recurrence. We have outlined a framework for describing this interaction between tumor cells left behind at surgery and the tissue surrounding the resection cavity during the period before or during adjuvant therapy. Immune suppression, as well as metabolic, oxidative, BBB disruption, debris and mechanical changes that occur in the resection site can all contribute to tumor regrowth. Engineered nanomedicine may play a role in treating GBM by providing a local, high-concentration depot of drugs at the tumor margin, but in addition to providing controlled drug release, realistic measurements of the human cavity, an honest account of the diffusion limits of local therapies, and more realistic models for predicting both safety and efficacy will be required. Local treatment of the margin(s) of highest risk for early infiltrating tumor cells and systemic therapy for possible distantly located, infiltrating tumor cells will likely form the core of most successful treatment strategies. Additionally, patient specific models for choosing between and combining different therapies will likely become the standard of care.

## Figures and Tables

**Table 1:** Comparison of the POCM with related concepts in the GBM literature

Feature dimension	POCM	SMe 10	Surgery-induced protumor microenvironment 30
Temporal definition	0 to 6 weeks after surgery, corresponding to the treatment gap and providing a quantifiable time window	Any postoperative time point, with a vague and nonquantifiable definition	Early to late postoperative period, with no clearly defined time range
Spatial definition	Physical resection cavity and the surrounding 2 to 5 mm marginal zone	No clear spatial boundary	Peritumoral region, poorly defined
Primary drivers	Surgical injury, including BBB disruption, NET formation, cellular debris, and reactive astrogliosis, together with residual tumor cells	Residual tumor cells	Surgery-induced inflammatory and repair signals
Quantifiable features	pH gradient, ROS/GSH levels, MMP overexpression, and mechanical instability	Few systematically quantifiable indicators	Selected inflammatory cytokines
Therapeutic significance	Defines a clear treatment gap for local intervention	Primarily descriptive, with limited guidance for treatment	Provides a warning by indicating protumor effects

**Table 2:** Distinguishing surgery-related differences between the POCM and the primary TME.

Feature	Primary TME	POCM after surgery	Reference
Physical structure	Intact tumor mass under solid stress	Physical cavity with altered hydrodynamic and mechanical states	
BBB	Chronic and heterogeneous disruption	Transient and quantifiable opening that peaks during the first week	28
NETs	Relatively sparse	Extensive NET formation around the resection cavity, promoting proliferation and migration of residual tumor cells	35
Reactive astrocytes	Chronic gliosis	Surgery-induced transition toward a protumor phenotype through Cxcl5 signaling	46
DAMP release	Tumor-driven and gradual	Burst release caused by extensive acute cell death	32
Lipid and metabolic signals	Chronic metabolic reprogramming	Acute myelin-debris burden, with LLM-mediated lipid recycling fueling mesenchymal-like GBM	56
Drivers of immune responses	Tumor-cell-intrinsic signals	Surgical injury, with wound-healing signals exploited by residual tumor cells	9

**Table 3:** Representative clinical trials of postoperative local therapy for glioma.

Candidate product	Technology platform	Indication	Clinical stage	ClinicalTrials.gov identifier	Current status or key result
Gliadel® Wafer (BCNU)	BCNU sustained-release implant	Newly diagnosed or recurrent malignant glioma	Approved, FDA 1996/2003	-	Limited survival benefit
GammaTile	Collagen-matrix radioactive seed implant	Newly diagnosed GBM	BRIDGES phase III trial	NCT07195591	First patient enrolled in January 2026
Irinotecan-ChemoSeed	Biodegradable sustained-release irinotecan implant	Resectable GBM	Phase II, approved by MHRA/REC and scheduled to begin in 2026	NCT07356973	Bypasses the BBB and targets the resection margin
Cerebraca® Wafer	Locally implanted investigational drug	Newly diagnosed GBM	Phase I/IIa completed, with phase IIb/III planned	NCT07349693	Overall mOS 15.7 months and 26.2 months in the RTK-high subgroup
CLD-101	Neural stem cells carrying an oncolytic virus	Recurrent high-grade glioma	Phase I	-	Fourteen patients treated with favorable safety
GLIORA (Nanoform/Revio)	Thermosensitive hydrogel containing olaparib and TMZ	High-grade glioma	Preclinical, with phase I expected in the second half of 2026	-	GMP prototype prepared

## Data Availability

The datasets used and analyzed in this study are available from the corresponding author upon reasonable request.

## Abbreviations

BBB: Blood-brain barrier
CAR-Ms: Chimeric antigen receptor macrophages
CDT: Chemodynamic therapy
CTL: Cytotoxic T lymphocyte
DAMPs: Damage-associated molecular patterns
ECM: Extra-cellular matrix
GBM: Glioblastoma
GSCs: Glioma stem cells
ICD: Immunogenic cell death
LLMs: Lipid-laden macrophages
MDMs: Monocyte-derived macrophages
PDT: Photodynamic therapy
POCM: Postoperative cavity microenvironment
RCD: Regulated cell death
ROS: Reactive oxygen species
sHDL: synthetic high-density lipoprotein
TAMs: Tumor-associated macrophages
TLR: Toll-like receptor

## References

[1] Price M, Ballard CAP, Benedetti JR, Kruchko C, Barnholtz-Sloan JS, Ostrom QT (2025). CBTRUS statistical report: pediatric brain tumor foundation childhood and adolescent primary brain and other central nervous system tumors diagnosed in the United states States in 2017-2021. Neuro Oncol.

[2] Zhu M, Yang M, Li R, Hu X, Chen J, Xu L (2025). Local therapeutic platform prevents postsurgical GBM recurrence by diminishing GICs and reshaping immunosuppressive microenvironment. Nat Commun.

[3] Sipos D, Raposa BL, Freihat O, Simon M, Mekis N, Cornacchione P (2025). Glioblastoma: clinical presentation, multidisciplinary management, and long-term outcomes. Cancers (Basel).

[4] Sughrue ME, Sheean T, Bonney PA, Maurer AJ, Teo C (2015). Aggressive repeat surgery for focally recurrent primary glioblastoma: outcomes and theoretical framework. Neurosurg Focus.

[5] Bastola S, Pavlyukov MS, Yamashita D, Ghosh S, Cho H, Kagaya N (2020). Glioma-initiating cells at tumor edge gain signals from tumor core cells to promote their malignancy. Nat Commun.

[6] Wang Z, Zhang H, Xu S, Liu Z, Cheng Q (2021). The adaptive transition of glioblastoma stem cells and its implications on treatments. Signal Transduct Target Ther.

[7] Hamard L, Ratel D, Selek L, Berger F, van der Sanden B, Wion D (2016). The brain tissue response to surgical injury and its possible contribution to glioma recurrence. J Neurooncol.

[8] Tini P, Donnini F, Rubino G, Battaglia G, Pastina P, Vannini M (2026). Beyond resection: surgery as an evolutionary bottleneck shaping tumor evolution and treatment response in diffuse gliomas. Cancers (Basel).

[9] Salmeron-Moreno K, Papisetty K, Kim CD, Pradilla G, Garzon-Muvdi T (2026). Neuro-oncological perspectives on cancer stem cell biology in glioblastoma: implications for resection, recurrence, targeted therapy, and other CNS tumors. Cells.

[10] Bastiancich C, Snacel-Fazy E, Fernandez S, Robert S, Stacchini R, Plantureux L (2024). Tailoring glioblastoma treatment based on longitudinal analysis of post-surgical tumor microenvironment. J Exp Clin Cancer Res.

[11] Brooks LJ, Simpson Ragdale H, Hill CS, Clements M, Parrinello S (2022). Injury programs shape glioblastoma. Trends Neurosci.

[12] Chen Z, Sang L, Zhai Q, Li X, Liu Y, Bai Z (2025). Ultrasound-responsive nanoparticles for imaging and therapy of brain tumors. Mater Today Bio.

[13] Muhammad S, Maridevaru MC, Roy S, Chen D, Zeng W, Sun L (2025). Piezodynamic therapy: unleashing mechanical energy and featuristic next generation therapeutic paradigms for glioblastoma. ACS Nano.

[14] Zam A, Rouatbi N, Walters AA, Al-Jamal KT (2026). Overcoming barriers and shaping the future: challenges and innovations in nucleic acid therapies for glioblastoma. Adv Drug Deliv Rev.

[15] Yang J, Shi Z, Liu R, Wu Y, Zhang X (2020). Combined-therapeutic strategies synergistically potentiate glioblastoma multiforme treatment via nanotechnology. Theranostics.

[16] Cui T, Chen S, Liu S, Niu X, Wang J, Ao R (2026). A biohybrid chiral hydrogel enhances preclinical postoperative glioblastoma therapy by multi-pronged inhibition of tumour stemness. Nat Nanotechnol.

[17] Dong Y, Zhang J, Wang Y, Zhang Y, Rappaport D, Yang Z (2024). Intracavitary spraying of nanoregulator-encased hydrogel modulates cholesterol metabolism of glioma-supportive macrophage for postoperative glioblastoma immunotherapy. Adv Mater.

[18] Kang T, Cha GD, Park OK, Cho HR, Kim M, Lee J (2023). Penetrative and sustained drug delivery using injectable hydrogel nanocomposites for postsurgical brain tumor treatment. ACS Nano.

[19] Liu R, Liang Q, Luo JQ, Li YX, Zhang X, Fan K (2024). Ferritin-based nanocomposite hydrogel promotes tumor penetration and enhances cancer chemoimmunotherapy. Adv Sci (Weinh).

[20] Shang Q, Jiang C, Guo M, Tang M, Yang J, Xie J (2026). STING stimulation via supramolecular prodrug hydrogel boosts innate-adaptive immune cross-talk to prevent glioblastoma recurrence. Sci Adv.

[21] Ye L, Lv W, He W, Li S, Min Z, Gong L (2023). Reduced malignant glioblastoma recurrence post-resection through the anti-CD47 antibody and temozolomide co-embedded in-situ hydrogel system. J Control Release.

[22] Zhao M, Bozzato E, Joudiou N, Ghiassinejad S, Danhier F, Gallez B (2019). Codelivery of paclitaxel and temozolomide through a photopolymerizable hydrogel prevents glioblastoma recurrence after surgical resection. J Control Release.

[23] Wu H, Cao P, Wang H, Wang W, Yu H, You C (2024). Postoperative injection of a triptolide-preloaded hydrogel prevents the recurrence of glioblastoma by dual-pathway activation of ferroptosis. Small.

[24] Zhang S, Zhong R, Younis MR, He H, Xu H, Li G (2024). Hydrogel applications in the diagnosis and treatment of glioblastoma. ACS Appl Mater Interfaces.

[25] Majumder P, Baxa U, Walsh STR, Schneider JP (2018). Design of a multicompartment hydrogel that facilitates time-resolved delivery of combination therapy and synergized killing of glioblastoma. Angew Chem Int Ed Engl.

[26] Chen S, Qiu Q, Wang D, She D, Yin B, Gu G (2022). Dual-sensitive drug-loaded hydrogel system for local inhibition of post-surgical glioma recurrence. J Control Release.

[27] Wang F, Huang Q, Su H, Sun M, Wang Z, Chen Z (2023). Self-assembling paclitaxel-mediated stimulation of tumor-associated macrophages for postoperative treatment of glioblastoma. Proc Natl Acad Sci U S A.

[28] Fernandes LF, Peeyatu C, Dickie BR, Ho YS, Thompson LA, Hernandez N (2025). Targeting therapeutic nanoparticles to the glioblastoma resection margin by harnessing post-operative spatiotemporal blood-brain barrier disruption. bioRxiv [Preprint].

[29] Liu F, Xu XH, Li CY, Zhang TT, Yin SL, Liu GQ (2021). Rapid tumor recurrence in a novel murine GBM surgical model is associated with Akt/PD-L1/vimentin signaling. Biochem Biophys Res Commun.

[30] Cheng X, Zhang H, Hamad A, Huang H, Tsung A (2022). Surgery-mediated tumor-promoting effects on the immune microenvironment. Semin Cancer Biol.

[31] de Los Reyes V AA, Jung E, Kim Y (2015). Optimal control strategies of eradicating invisible glioblastoma cells after conventional surgery. J R Soc Interface.

[32] Kim Y, Lee D, Lawler S (2020). Collective invasion of glioma cells through OCT1 signalling and interaction with reactive astrocytes after surgery. Philos Trans R Soc Lond B Biol Sci.

[33] Zhang Y, Xi K, Fu Z, Zhao X, Zhang Q, Fu H (2024). Stimulation of tumoricidal immunity via bacteriotherapy inhibits glioblastoma relapse. Nat Commun.

[34] Buonfiglioli A, Hambardzumyan D (2021). Macrophages and microglia: the cerberus of glioblastoma. Acta Neuropathol Commun.

[35] Han Y, Han M, Wang T, Zhang H, Liu H, Zheng Y (2025). Inhibiting the formation of neutrophil extracellular traps to prevent the recurrence of post-operative glioblastoma. Nat Commun.

[36] Wang ZD, Geng HM, Zhang YQ, Liu YF, Wang Y, Chen Y (2023). Lymph node-inspired immunoregulatory hydrogel with siRNA delivery property for postoperative glioblastoma treatment. Chem Eng J.

[37] Chen C, Jing W, Chen Y, Wang G, Abdalla M, Gao L (2022). Intracavity generation of glioma stem cell-specific CAR macrophages primes locoregional immunity for postoperative glioblastoma therapy. Sci Transl Med.

[38] Fu Z, Zhao X, Zhang Q, Liu Y, Zhang Y, Li Z (2026). Synthetic SIGLEC9-based chimeric switch receptor augments the efficacy of CAR macrophages against glioblastoma. Proc Natl Acad Sci U S A.

[39] Tian Y, Liu C, Li Z, Wang Y, Zhang X, Chen H (2022). Exosomal B7-H4 from irradiated glioblastoma cells contributes to increase FoxP3 expression of differentiating Th1 cells and promotes tumor growth. Redox Biol.

[40] De Leo A, Ugolini A, Yu X, Sciacca R, Pricoco G, Battaglia G (2024). Glucose-driven histone lactylation promotes the immunosuppressive activity of monocyte-derived macrophages in glioblastoma. Immunity.

[41] Chen J, Huang Z, Chen Y, Li Y, Zhao Y, Zhang S (2025). Lactate and lactylation in cancer. Signal Transduct Target Ther.

[42] Colegio OR, Chu NQ, Szabo AL, Chu T, Rhebergen AM, Jairam V (2014). Functional polarization of tumour-associated macrophages by tumour-derived lactic acid. Nature.

[43] Wang S, Huang T, Wu Q, Li M, Zhang Y, Chen J (2024). Lactate reprograms glioblastoma immunity through CBX3-regulated histone lactylation. J Clin Invest.

[44] Li D, Cui G, Yang K, Wang Y, Liu X, Zhang L (2026). Inhibiting macrophage-derived lactate transport restores cGAS-STING signalling and enhances antitumour immunity in glioblastoma. Nat Cell Biol.

[45] Wang S, Yan W, Kong L, Zhang Y, Li M, Chen J (2023). Oncolytic viruses engineered to enforce cholesterol efflux restore tumor-associated macrophage phagocytosis and anti-tumor immunity in glioblastoma. Nat Commun.

[46] Kloosterman DJ, Erbani J, Boon M, Bongaarts A, de Bruijn L, de Bruin R (2024). Macrophage-mediated myelin recycling fuels brain cancer malignancy. Cell.

[47] Tian Z, Chen Y, Zhou J, Li W, Wang H, Zhang L (2026). A supramolecular nanoscavengers: based "cargo-exchange" breaking cholesterol metabolic dysregulation for glioblastoma therapy. Adv Sci (Weinh).

[48] Qu D, Bai Y, Liu X, Wang Y, Zhang H, Li S (2026). Biodegradable nano-regulator reprograms glioblastoma immunosuppression: pyroptosis-metabolism-immunity crosstalk for cascading immune activation. Angew Chem Int Ed Engl.

[49] Halseth TA, Mujeeb AA, Liu LS, Zhang Y, Chen J, Wang F (2025). HDL nanodiscs loaded with liver X receptor agonist decreases tumor burden and mediates long-term survival in mouse glioma model. Small.

[50] Kim NY, Gowda SGS, Lee SG, Park JH, Kim HY, Kim MJ (2024). Cannabidiol induces ERK activation and ROS production to promote autophagy and ferroptosis in glioblastoma cells. Chem Biol Interact.

[51] Su IC, Su YK, Setiawan SA, Wang HC, Chen YC, Chen CC (2023). NADPH oxidase subunit CYBB confers chemotherapy and ferroptosis resistance in mesenchymal glioblastoma via Nrf2/SOD2 modulation. Int J Mol Sci.

[52] Dixon SJ, Olzmann JA (2024). The cell biology of ferroptosis. Nat Rev Mol Cell Biol.

[53] Zhou Q, Meng Y, Li D, Wang Y, Zhang X, Liu Z (2024). Ferroptosis in cancer: from molecular mechanisms to therapeutic strategies. Signal Transduct Target Ther.

[54] Qiu C, Zhang X, Huang B, Wang Y, Li J, Chen H (2020). Disulfiram, a ferroptosis inducer, triggers lysosomal membrane permeabilization by up-regulating ROS in glioblastoma. Onco Targets Ther.

[55] Yang L, Jiang Z, Huang L, Wang Y, Zhang X, Chen J (2026). Disrupting copper homeostasis to enhance cuproptosis and ferroptosis for glioblastoma immunotherapy. Adv Healthc Mater.

[56] Pagano Zottola AC, Daubon T, Venkataramani V (2024). Inside help for brain tumors: macrophage-mediated myelin recycling promotes cell state-specific glioblastoma progression. Signal Transduct Target Ther.

[57] Krishnamachari Y, Salem AK (2009). Innovative strategies for co-delivering antigens and CpG oligonucleotides. Adv Drug Deliv Rev.

[58] Weiner GJ (2009). CpG oligodeoxynucleotide-based therapy of lymphoid malignancies. Adv Drug Deliv Rev.

[59] Zhang Y, You H, Wang Y, Li X, Chen J, Zhang L (2022). A micro-environment regulator for filling the clinical treatment gap after a glioblastoma operation. Adv Healthc Mater.

[60] Cui J, Wang X, Li J, Zhang Y, Chen J, Liu S (2023). Immune exosomes loading self-assembled nanomicelles traverse the blood-brain barrier for chemo-immunotherapy against glioblastoma. ACS Nano.

[61] Kang XY, Chen WW, Zhang Y, Li H, Wang F, Liu Y (2026). A sono-responsive nanoplatform integrating STING activation and CXCR4 blockade for synergistic immunotherapy of glioblastoma. Adv Mater.

[62] Meng JH, Chen MC, Gu F, Wang Y, Zhang L, Li X (2025). *In situ* formed chemo-immunotherapeutic hydrogel for suppression of postoperative glioma recurrence and intraoperative hemostasis. J Control Release.

[63] Sun S, Gu W, Wu H, Zhao Q, Qian S, Xiao H (2022). Immunostimulant *in situ* hydrogel improves synergetic radioimmunotherapy of malignant glioblastoma relapse post-resection. Adv Funct Mater.

[64] Mantovani A, Allavena P, Marchesi F, Garlanda C (2022). Macrophages as tools and targets in cancer therapy. Nat Rev Drug Discov.

[65] Yamada-Hunter SA, Theruvath J, McIntosh BJ, Zhang Y, Chen J, Wang F (2024). Engineered CD47 protects T cells for enhanced antitumour immunity. Nature.

[66] Zhou L, Song Q, Zhang X, Wang Y, Li M, Chen H (2025). *In vivo* generation of CAR macrophages via the enucleated mesenchymal stem cell delivery system for glioblastoma therapy. Proc Natl Acad Sci U S A.

[67] Ying K, Zhu Y, Wan J, Zhang X, Chen J, Wang F (2023). Macrophage membrane-biomimetic adhesive polycaprolactone nanocamptothecin for improving cancer-targeting efficiency and impairing metastasis. Bioact Mater.

[68] Luo JQ, Zhang J, Yu HH, Li YX, Fan K, Du JZ (2025). Genetically engineered biomimetic nanoparticles for synergistic activation of glioma-associated macrophages against glioblastoma. J Am Chem Soc.

[69] Guo Y, Jiang T, Liang S, Wang Y, Zhang L, Chen J (2025). Immunostimulatory hydrogel with synergistic blockage of glutamine metabolism and chemodynamic therapy for postoperative management of glioblastoma. Adv Sci (Weinh).

[70] Guo Y, Jin L, Shen Z, Wang Y, Zhang X, Chen H (2025). Biomimetic membrane vesicles reprogram microglia polarization and remodel the immunosuppressive microenvironment of glioblastoma via PERK/HIF-1α/glycolysis pathway. Adv Healthc Mater.

[71] Han X, Feng F, Wang Z, Zhang Y, Chen J, Li M (2026). Preventing glioblastoma relapse by igniting innate immunity through mitochondrial stress in the surgical cavity. Adv Mater.

[72] Krysko DV, Garg AD, Kaczmarek A, Krysko O, Agostinis P, Vandenabeele P (2012). Immunogenic cell death and DAMPs in cancer therapy. Nat Rev Cancer.

[73] Yang JH, Zhang K, Zhang GL, Wang Y, Chen J, Li X (2025). Autologous nanovaccine induces immunogenic domino effect to prevent postoperative recurrence of orthotopic glioblastoma. Adv Funct Mater.

[74] Zhang C, Hou S, Hu B, Wang Y, Zhang L, Chen J (2025). Self-assembling nanoparticles orchestrate cuproptosis-immunotherapy synergy to suppress postoperative glioma recurrence. ACS Appl Mater Interfaces.

[75] Bao P, Gu HY, Jiang YC, Wang Y, Zhang X, Chen H (2024). In situ sprayed exosome-cross-linked gel as artificial lymph nodes for postoperative glioblastoma immunotherapy. ACS Nano.

[76] Yalamandala BN, Moorthy T, Liu ZH, Wang Y, Chen J, Li M (2025). A self-cascading catalytic therapy and antigen capture scaffold-mediated T cells augments for postoperative brain immunotherapy. Small.

[77] Zhang RT, Song YZ, Yao JW, Wang Y, Chen J, Li M (2025). RNA-Seq reveals the mechanism of synergistic hydrogen-chemotherapy based on active magnesium micromotors for inhibiting glioblastoma recurrence by modulating tumor microenvironment. Small.

[78] Mbah NE, Sutton D, Hong HS, Zhang Y, Chen J, Wang F (2026). Tumor cell death by ferroptosis contributes to an immunosuppressive tumor microenvironment in syngeneic murine models of cancer. Cancer Metab.

[79] Minton K (2026). Ferroptotic tumour cells inhibit dendritic cell maturation through GPX4 release. Nat Rev Immunol.

[80] Castillo JG, Silveria S, Schirokauer L, Wang Y, Zhang X, Chen H (2026). Selective disruption of lipid peroxide homeostasis in intratumoral regulatory T cells by targeting FSP1 enhances cancer immunity. Sci Adv.

[81] Pathan-Chhatbar S, Drechsler C, Richter K, Tampé R (2021). Direct regulation of the T cell antigen receptor's activity by cholesterol. Front Cell Dev Biol.

[82] Huang TX, Huang HS, Dong SW, Wang Y, Zhang L, Chen J (2024). ATP6V0A1-dependent cholesterol absorption in colorectal cancer cells triggers immunosuppressive signaling to inactivate memory CD8⁺ T cells. Nat Commun.

[83] Lee J, Jallo GI, Penno MB, Gabrielson KL (2006). Intracranial drug-delivery scaffolds: biocompatibility evaluation of sucrose acetate isobutyrate gels. Toxicol Appl Pharmacol.

[84] Tamargo RJ, Epstein JI, Reinhard CS, Chasin M, Brem H (1989). Brain biocompatibility of a biodegradable, controlled-release polymer in rats. J Biomed Mater Res.

[85] Gao J, Song Q, Gu X, Jiang G, Huang J, Tang Y (2024). Intracerebral fate of organic and inorganic nanoparticles is dependent on microglial extracellular vesicle function. Nat Nanotechnol.

[86] Bjugstad KB, Lampe K, Kern DS, Mahoney M (2010). Biocompatibility of poly(ethylene glycol)-based hydrogels in the brain: an analysis of the glial response across space and time. J Biomed Mater Res A.

[87] Chen D, Yu C, Xia S, Wang Y, Zhang X, Chen H (2026). Enhanced immunotherapy for glioblastoma using a cholesterol oxidase-loaded, Pd-doped Cu₃N nanozyme-based multimodal nanoplatform. ACS Nano.

[88] Zhang Y, Fu X, Jia J, Wang Y, Chen J, Li M (2020). Glioblastoma therapy using codelivery of cisplatin and glutathione peroxidase targeting siRNA from iron oxide nanoparticles. ACS Appl Mater Interfaces.

[89] Liu S, Sun K, Li M, Wang Y, Zhang L, Chen J (2025). Quadruple synergistic amplification of ferroptosis for precision glioblastoma therapy: a luteolin-coordinated ferric ion nanoplatform. J Nanobiotechnology.

[90] Gai S, Yan Q, Li S, Wang Y, Zhang X, Chen H (2025). Lactoferrin nanoparticle-vanadium complex: a promising high-efficiency agent against glioblastoma by triggering autophagy and ferroptosis. J Med Chem.

[91] Jia M, Zhou X, Li P, Wang Y, Zhang L, Chen J (2024). An injectable biomimetic hydrogel adapting brain tissue mechanical strength for postoperative treatment of glioblastoma without anti-tumor drugs participation. J Control Release.

[92] Zhang X, Tang B, Luo J, Wang Y, Chen H, Li M (2024). Cuproptosis, ferroptosis and PANoptosis in tumor immune microenvironment remodeling and immunotherapy: culprits or new hope. Mol Cancer.

[93] Xue Q, Yan D, Chen X, Wang Y, Zhang L, Chen J (2023). Copper-dependent autophagic degradation of GPX4 drives ferroptosis. Autophagy.

[94] Feng W, Weng C, Li C, Wang Y, Zhang X, Chen H (2026). Omeprazole-mediated nanodelivery of copper for synchronous remodeling of copper influx and efflux in cuproptotic glioblastoma therapy. Acta Biomater.

[95] Liu MM, Zhao LX, Gong ZQ, Wang Y, Chen J, Li M (2025). Engineered RAP-anchored copper-escorting liposomes for FDX1-targeted cuproptosis in glioblastoma therapy. Theranostics.

[96] Liu Y, Wu Y, Cao R, Wang Y, Zhang L, Chen J (2026). A "depolarization-activation" strategy targeting microglia: cuproptosis-mediated immune re-sensitization nanoplatform in glioma therapy. Adv Funct Mater.

[97] Gu X, Song Q, Zhang Q, Huang M, Zheng M, Chen J (2020). Clearance of two organic nanoparticles from the brain via the paravascular pathway. J Control Release.

[98] Airan RD (2020). Glymphatics rapidly clear nanoparticles. Sci Transl Med.

[99] Guo H, Wei Z, Zhou H, Liu Z, Yuan T, Wang T (2025). Microinvasive deployment and fate-determination of functional, engineered nanoparticles in central nervous system. ACS Materials Letters.

[100] Lv R, Li F, Liu Y, Song M, Yuan J, Zhang G (2025). Molecularly imprinted nanoparticles hitchhiking on neutrophils for precise treatment of ischemic stroke. J Colloid Interface Sci.

[101] Hu K, Liu Y, Wang Q, Xiong Y, Guo Z, Weng Z (2023). Copper nanoclusters based short-term memory "eraser". Chem Eng J.

[102] Hou SQ, Zhang C, Wang Y, Chen J, Li M, Zhang L (2026). Glioma cell membrane-coated CaCO₃ nanoparticles for localized postoperative chemo-calcium overload therapy to prevent glioma recurrence. Int J Nanomedicine.

[103] Zhu YF, Jia J, Zhao G, Wang Y, Zhang X, Chen H (2021). Multi-responsive nanofibers composite gel for local drug delivery to inhibit recurrence of glioma after operation. J Nanobiotechnology.

[104] Kuang Y, Zhang Z, Zhu K, Wang Y, Chen J, Li M (2024). Porphyrin-based-MOF nanocomposite hydrogels for synergistic sonodynamic and gas therapy against tumor. Int J Biol Macromol.

[105] Barbugian F, Salerno D, Ballarini E, Wang Y, Zhang L, Chen J (2025). Bioresponsive hyaluronic acid-based hydrogel inhibits matrix metalloproteinase-2 in glioblastoma microenvironment. ChemMedChem.

[106] Xue DZ, Cao Y, Li WY, Wang Y, Zhang X, Chen H (2026). Sequential programmed nanomachine to optimize NIR-II fluorescence guided surgery and immune amplification for orthotopic glioblastoma. Chem Eng J.

[107] Cao X, Li SN, Chen WL, Wang Y, Chen J, Li M (2022). Multifunctional hybrid hydrogel system enhanced the therapeutic efficacy of treatments for postoperative glioma. ACS Appl Mater Interfaces.

[108] Nie D, Ling Y, Lv W, Wang Y, Zhang L, Chen J (2023). *In situ* attached photothermal immunomodulation-enhanced nanozyme for the inhibition of postoperative malignant glioma recurrence. ACS Nano.

[109] Chen Y, Ma Y, Shi K, Wang Y, Zhang X, Chen H (2024). Self-disassembling and oxygen-generating porphyrin-lipoprotein nanoparticle for targeted glioblastoma resection and enhanced photodynamic therapy. Adv Mater.

[110] Huang CY, Yang HW, Wang HC, Wang Y, Chen J, Li M (2025). Gelatin-based rapid blue light-irradiation *in situ* gelation hydrogel platform for combination therapy in brain tumors. Pharmaceutics.

[111] Isik S, Yucel D, Hasirci V (2025). Development of a hydrogel platform with GBM and microglia: a potential glioblastoma tumor model. ACS Appl Bio Mater.

[112] Schroyer KAR, Schmitz KM, Raheja G, Su B, Lathia JD, Ning L (2025). Bioprinting of GelMA-based hydrogels to aid in creation of biomimetic 3D models for glioblastoma. Micromachines (Basel).

[113] Sinha S, Fleck M, Ayushman M, Tong X, Mikos G, Jones S (2025). Matrix stiffness regulates GBM migration and chemoradiotherapy responses via chromatin condensation in 3D viscoelastic matrices. ACS Appl Mater Interfaces.

[114] Bomb K, Zhang Q, Ford EM, Fromen CA, Kloxin AM (2023). Systematic d-amino acid substitutions to control peptide and hydrogel degradation in cellular microenvironments. ACS Macro Lett.

[115] Kou JH, Emmett C, Shen P, Aswani S, Iwamoto T, Vaghefi F (1997). Bioerosion and biocompatibility of poly(d,l-lactic-co-glycolic acid) implants in brain. J Control Release.

[116] Haile Y, Berski S, Dräger G, Nobre A, Stummeyer K, Gerardy-Schahn R (2008). The effect of modified polysialic acid based hydrogels on the adhesion and viability of primary neurons and glial cells. Biomaterials.

[117] Kamkin A, Kiseleva I (2009). Mechanosensitivity of the nervous system. Dordrecht: Springer.

[118] Wakimoto H, Mohapatra G, Kanai R, Curry WT Jr, Yip S, Nitta M (2012). Maintenance of primary tumor phenotype and genotype in glioblastoma stem cells. Neuro Oncol.

[119] Tejero R, Huang Y, Katsyv I, Kluge M, Lin JY, Tome-Garcia J (2019). Gene signatures of quiescent glioblastoma cells reveal mesenchymal shift and interactions with niche microenvironment. EBioMedicine.

[120] Branco F, Cunha J, Mendes M, Wang Y, Zhang L, Chen J (2025). 3D bioprinting models for glioblastoma: from scaffold design to therapeutic application. Adv Mater.

[121] Reátegui E, van der Vos KE, Lai CP, Zeinali M, Atai NA, Dekker B (2018). Engineered nanointerfaces for microfluidic isolation and molecular profiling of tumor-specific extracellular vesicles. Nat Commun.

[122] Tang M, Rich JN, Chen S (2021). Biomaterials and 3D bioprinting strategies to model glioblastoma and the blood-brain barrier. Adv Mater.

[123] Tang M, Xie Q, Gimple RC, Zhong Z, Tam T, Tian J (2020). Three-dimensional bioprinted glioblastoma microenvironments model cellular dependencies and immune interactions. Cell Res.

[124] Li X, Zhao F, Xu S, Wang Y, Zhang L, Chen J (2026). Patient-derived 3D bioprinted glioblastoma models with defined physicochemical ECM properties for long-term maintenance and CAR-T therapy evaluation. Chem Eng J.

[125] Tripathy A, Ji S, Serhan H, Wang Y, Chen J, Li M (2025). Precision oncology for high-grade gliomas: a tumor organoid model for adjuvant treatment selection. Bioengineering (Basel).

[126] Camenisch S, Bell L, Stokar-Regenscheit N, Wang Y, Zhang X, Chen H (2025). GliaMimic: a longitudinal, multimodal *in vitro* platform for evaluating glioblastoma treatment response and relapse in patient-derived organoids or spheroids. bioRxiv.

[127] Pun S, Prakash A, Demaree D, Wang Y, Chen J, Li M (2024). Rapid biofabrication of an advanced microphysiological system mimicking phenotypical heterogeneity and drug resistance in glioblastoma. Adv Healthc Mater.

[128] Hariri A, Zarepour A, Khosravi A, Mirian M, Iravani S, Zarrabi A (2025). Engineering vascularized brain tumor organoids: bridging the gap between models and reality. Biomed Microdevices.

[129] Seyfoori A, Liu K, Caruncho HJ, Wang Y, Chen J, Li M (2025). Tumoroid-on-a-plate (ToP): physiologically relevant cancer model generation and therapeutic screening. Adv Healthc Mater.

[130] Bezze A, Muccio S, Ciardelli G, Wang Y, Zhang L, Chen J (2025). Engineering human-relevant glioblastoma microenvironment models for the optimization of advanced drug delivery systems. ALTEX Proc.

[131] Manoharan TJM, Wang TY, Mantri S, Wang Y, Chen J, Li M (2026). Advancing targeted drug delivery in glioblastoma multiforme through biomimetic nanomedicine using 3D tumor-on-a-chip model. Adv Healthc Mater.

[132] Youngblood MW, Kumari A, Kang YT, Wang Y, Zhang X, Chen H (2025). Dynamic release of extracellular particles after opening of the blood-brain barrier predicts glioblastoma susceptibility to paclitaxel. Nat Commun.

[133] Zheng C, Wang P, Zhang D, Wang Y, Chen J, Li M (2025). A novel organoid model retaining the glioma microenvironment for personalized drug screening and therapeutic evaluation. Bioact Mater.

[134] Zhu G, Shi X, Jiang Y, Wang Y, Zhang L, Chen J (2025). Microgravity-cultured glioblastoma organoids integrated with microfluidic chip for CAR-γδ T evaluation. Commun Biol.

[135] Taleban H, Li X, Ali Z, Wang Y, Chen J, Li M (2026). Integrating computational fluid dynamics into organ-on-chip systems: a glioblastoma-centred design and validation framework. Front Bioeng Biotechnol.

[136] Butler D (2008). Translational research: crossing the valley of death. Nature.

[137] Bastiancich C, Malfanti A, Préat V, Danhier F (2021). Rationally designed drug delivery systems for the local treatment of resected glioblastoma. Adv Drug Deliv Rev.

[138] Wang G, Zhong K, Wang Z, Zhang Y, Chen J, Li M (2022). Tumor-associated microglia and macrophages in glioblastoma: from basic insights to therapeutic opportunities. Front Immunol.

[139] van der Meel R, Fens MH, Vader P, van Solinge WW, Eniola-Adefeso O, Schiffelers RM (2014). Extracellular vesicles as drug delivery systems: lessons from the liposome field. J Control Release.

[140] Brem H, Piantadosi S, Burger PC, Walker M, Selker R, Vick NA (1995). Placebo-controlled trial of safety and efficacy of intraoperative controlled delivery by biodegradable polymers of chemotherapy for recurrent gliomas. Lancet.

[141] Westphal M, Hilt DC, Bortey E, Delavault P, Olivares R, Warnke PC (2003). A phase 3 trial of local chemotherapy with biodegradable carmustine (BCNU) wafers (Gliadel wafers) in patients with primary malignant glioma. Neuro Oncol.

[142] Hart MG, Grant R, Garside R, Rogers G, Somerville M, Stein K (2011). Chemotherapy wafers for high grade glioma. Cochrane Database Syst Rev.

[143] Bastiancich C, Bianco J, Vanvarenberg K, Ucakar B, Joudiou N, Gallez B (2017). Injectable nanomedicine hydrogel for local chemotherapy of glioblastoma after surgical resection. J Control Release.

[144] Tykocki T (2026). Immunotherapy failure in glioblastoma: a systematic review and meta-analysis of randomized controlled trials. Crit Rev Oncol Hematol.

[145] Reardon DA, Brandes AA, Omuro A, Mulholland P, Lim M, Wick A (2020). Effect of nivolumab vs bevacizumab in patients with recurrent glioblastoma: the CheckMate 143 phase 3 randomized clinical trial. JAMA Oncol.

[146] Omuro A, Brandes AA, Carpentier AF, Idbaih A, Reardon DA, Cloughesy T (2023). Radiotherapy combined with nivolumab or temozolomide for newly diagnosed glioblastoma with unmethylated MGMT promoter: an international randomized phase III trial. Neuro Oncol.

[147] Lim M, Weller M, Idbaih A, Steinbach J, Finocchiaro G, Raval RR (2022). Phase III trial of chemoradiotherapy with temozolomide plus nivolumab or placebo for newly diagnosed glioblastoma with methylated MGMT promoter. Neuro Oncol.

[148] Schonfeld E, Choi J, Tran A, Kim LH, Lim M (2024). The landscape of immune checkpoint inhibitor clinical trials in glioblastoma: a systematic review. Neurooncol Adv.

[149] Jaber M, Dzhodzhua L, Khan H, Abdelghani M, Aburas E (2025). The role of immune checkpoint inhibitors in the treatment of glioblastoma: a literature review of current evidence. Junior Researchers.

[150] Artzi SB, Klausen MN, Harwood DSL, Michaelsen SR, Maarup SB, Locallo A (2025). Spatial transcriptomic analysis reveals lack of response to PD-1 blockade in recurrent glioblastoma. Acta Neuropathol.

[151] Kim G, Lee J, Park JH, Kim KM, Khang D (2026). Multistage nanomedicine engineering to overcome sequential barriers to glioblastoma treatment: a review. J Nanobiotechnology.

[152] Khan ZM, Zhang J, Gannon J, Johnson BN, Verbridge SS, Vlaisavljevich E (2024). Development of an injectable hydrogel for histotripsy ablation toward future glioblastoma therapy applications. Ann Biomed Eng.

[153] Khan ZM, Wilts E, Vlaisavljevich E, Long TE, Verbridge SS (2022). Characterization and structure-property relationships of an injectable thiol-Michael addition hydrogel toward compatibility with glioblastoma therapy. Acta Biomater.

[154] Akpalo E, Larreta-Garde V (2010). Increase of fibrin gel elasticity by enzymes: a kinetic approach. Acta Biomater.

[155] Gandhi JK, Heinrich L, Knoff DS, Kim M, Marmorstein AD (2021). Alteration of fibrin hydrogel gelation and degradation kinetics through addition of azo dyes. J Biomed Mater Res A.

[156] Weisel JW, Litvinov RI (2013). Mechanisms of fibrin polymerization and clinical implications. Blood.

[157] Wei G, Xu H, Ding PT, Li SM, Zheng JM (2002). Thermosetting gels with modulated gelation temperature for ophthalmic use: the rheological and gamma scintigraphic studies. J Control Release.

[158] He Z, Meng Z, Luan G, Li P, Yang C, Liang Y (2026). Engineering glioma-cell-derived exosomes as Trojan horse for precisely targeted chemotherapy of glioblastoma. Biomater Res.

[159] Liu X, Zhang X, Fan Y, Wang B, Wang J, Zeng M (2025). Intraoperative goal-directed fluid management and postoperative brain edema in patients having high-grade gliomas resections: a randomized trial. Int J Surg.

[160] Suita Y, Miriyala S, Merih-Toruner D, Pizzagalli M, Leary OP, Yue W (2025). GliaTrap is a biodegradable, non-swelling and non-inflammatory hydrogel with tuned release of CXCL12 to attract migrating glioblastoma cells. Sci Rep.

[161] Nance EA, Woodworth GF, Sailor KA, Shih TY, Xu Q, Swaminathan G (2012). A dense poly(ethylene glycol) coating improves penetration of large polymeric nanoparticles within brain tissue. Sci Transl Med.

[162] Zhang C, Mastorakos P, Sobral M, Wang Y, Chen J, Li M (2017). Strategies to enhance the distribution of nanotherapeutics in the brain. J Control Release.

[163] Souweidane MM, Kramer K, Pandit-Taskar N, Wang Y, Zhang L, Chen J (2025). Phase 1 dose-escalation trial using convection-enhanced delivery of radio-immunotheranostic ¹²⁴I-Omburtamab for diffuse intrinsic pontine glioma. Neuro Oncol.

[164] van Solinge TS, Nieland L, Chiocca EA, Broekman MLD (2022). Advances in local therapy for glioblastoma - taking the fight to the tumour. Nat Rev Neurol.

[165] Hoopes PJ, Tavakkoli AD, Moodie KA, Wang Y, Chen J, Li M (2024). Porcine-human glioma xenograft model. Immunosuppression and model reproducibility. Cancer Treat Res Commun.

[166] Khoshnevis M, Carozzo C, Ponce F, Wang Y, Zhang L, Chen J (2017). Development of induced glioblastoma by implantation of a human xenograft in Yucatan minipig as a large animal model. J Neurosci Methods.

[167] Filley A, Henriquez M, Bhowmik T, Wang Y, Chen J, Li M (2018). Immunologic and gene expression profiles of spontaneous canine oligodendrogliomas. J Neurooncol.

[168] Hicks WH, Bird CE, Pernik MN, Wang Y, Zhang L, Chen J (2021). Large animal models of glioma: current status and future prospects. Anticancer Res.

[169] Ammons DT, Guth A, Rozental AJ, Wang Y, Chen J, Li M (2022). Reprogramming the canine glioma microenvironment with tumor vaccination plus oral losartan and propranolol induces objective responses. Cancer Res Commun.

[170] Hu Z, Luo J, Lou J, Deng J, Gong ZT, Chen J (2026). Neurosurgery as an immune anchor point: a translational framework for perioperative immunoengineering. Front Immunol.

[171] Mitchell D, Shireman J, Sierra Potchanant EA, Lara-Velazquez M, Dey M (2021). Neuroinflammation in autoimmune disease and primary brain tumors: the quest for striking the right balance. Front Cell Neurosci.

[172] Oriquat G, Abdulqader AF, Farid H, Ashurov Z, Sottarov A, Ghafl NY (2026). From protector to perpetrator: the cGAS-STING pathway at the intersection of neurodegeneration and neuroinflammation. Brain Res Bull.

[173] Hinkle JT, Patel J, Panicker N, Karuppagounder SS, Biswas D, Belingon B (2022). STING mediates neurodegeneration and neuroinflammation in nigrostriatal α-synucleinopathy. Proc Natl Acad Sci U S A.

[174] Butchi NB, Woods T, Du M, Morgan TW, Peterson KE (2011). TLR7 and TLR9 trigger distinct neuroinflammatory responses in the CNS. Am J Pathol.

[175] Zamyatina A, Heine H (2020). Lipopolysaccharide recognition in the crossroads of TLR4 and caspase-4/11 mediated inflammatory pathways. Front Immunol.

[176] Đorđević S, Gonzalez MM, Conejos-Sánchez I, Wang Y, Chen J, Li M (2022). Current hurdles to the translation of nanomedicines from bench to the clinic. Drug Deliv Transl Res.

[177] Anwar A, Sun P, Rong X, Wang Y, Zhang L, Chen J (2023). Process analytical technology as in-process control tool in semi-continuous manufacturing of PLGA/PEG-PLGA microspheres. Heliyon.

[178] Wang X, Chen Z, Wang Z, Wang Y, Chen J, Li M (2025). Toward preclinical evaluation of a thermosensitive PEG-poly(-alanine) hydrogel: a study on sterilization, storage stability, and in vivo performance. Acta Biomater.

[179] Dalwadi C, Patel G (2016). Implementation of "quality by design (QbD)" approach for the development of 5-fluorouracil loaded thermosensitive hydrogel. Curr Drug Deliv.

[180] Troiano G, Nolan J, Parsons D, Wang Y, Zhang L, Chen J (2016). A quality by design approach to developing and manufacturing polymeric nanoparticle drug products. AAPS J.

[181] De Lauretis A, Eriksson Agger A, Pal A, Wang Y, Chen J, Li M (2025). Balancing sterilization and functional properties in Poloxamer 407 hydrogels: comparing heat and radiation techniques. Regen Biomater.

[182] Wang K, Sun J, Zhao H, Wang Y, Zhang L, Chen J (2025). Advances and challenges in nano-delivery systems for glioblastoma treatment: a comprehensive review. Int J Nanomedicine.

[183] Riccobelli P, Pierce BF, Craparo EF, Wang Y, Chen J, Li M (2025). Advances in nanoparticle systems for targeted therapy against glioblastoma multiforme. Int J Pharm.

[184] Lee JW, Park JH, Yu GW, Wang Y, Zhang L, Chen J (2025). Sustained-release intra-articular drug delivery: PLGA systems in clinical context and evolving strategies. Pharmaceutics.

